# Taxonomic and functional stability overrules seasonality in polar benthic microbiomes

**DOI:** 10.1093/ismejo/wrad005

**Published:** 2024-01-10

**Authors:** Sebastian Miksch, Luis H Orellana, Monike Oggerin de Orube, Silvia Vidal-Melgosa, Vipul Solanki, Jan-Hendrik Hehemann, Rudolf Amann, Katrin Knittel

**Affiliations:** Department of Molecular Ecology, Max Planck Institute for Marine Microbiology, 28359 Bremen, Germany; Department of Molecular Ecology, Max Planck Institute for Marine Microbiology, 28359 Bremen, Germany; Department of Molecular Ecology, Max Planck Institute for Marine Microbiology, 28359 Bremen, Germany; Department of Molecular Ecology, Max Planck Institute for Marine Microbiology, 28359 Bremen, Germany; MARUM MPG Bridge Group Marine Glycobiology, Center for Marine Environmental Sciences, University of Bremen, 28359 Bremen, Germany; Department of Molecular Ecology, Max Planck Institute for Marine Microbiology, 28359 Bremen, Germany; Department of Molecular Ecology, Max Planck Institute for Marine Microbiology, 28359 Bremen, Germany; MARUM MPG Bridge Group Marine Glycobiology, Center for Marine Environmental Sciences, University of Bremen, 28359 Bremen, Germany; Department of Molecular Ecology, Max Planck Institute for Marine Microbiology, 28359 Bremen, Germany; Department of Molecular Ecology, Max Planck Institute for Marine Microbiology, 28359 Bremen, Germany

**Keywords:** marine sediment, glycan degradation, microbial diversity, metatranscriptomics, rRNA, Colwellia, Svalbard, Isfjorden

## Abstract

Coastal shelf sediments are hot spots of organic matter mineralization. They receive up to 50% of primary production, which, in higher latitudes, is strongly seasonal. Polar and temperate benthic bacterial communities, however, show a stable composition based on comparative 16S rRNA gene sequencing despite different microbial activity levels. Here, we aimed to resolve this contradiction by identifying seasonal changes at the functional level, in particular with respect to algal polysaccharide degradation genes, by combining metagenomics, metatranscriptomics, and glycan analysis in sandy surface sediments from Isfjorden, Svalbard. Gene expressions of diverse carbohydrate-active enzymes changed between winter and spring. For example, β-1,3-glucosidases (e.g. GH30, GH17, GH16) degrading laminarin, an energy storage molecule of algae, were elevated in spring, while enzymes related to α-glucan degradation were expressed in both seasons with maxima in winter (e.g. GH63, GH13_18, and GH15). Also, the expression of GH23 involved in peptidoglycan degradation was prevalent, which is in line with recycling of bacterial biomass. Sugar extractions from bulk sediments were low in concentrations during winter but higher in spring samples, with glucose constituting the largest fraction of measured monosaccharides (84% ± 14%). In porewater, glycan concentrations were ~18-fold higher than in overlying seawater (1107 ± 484 vs. 62 ± 101 μg C l^−1^) and were depleted in glucose. Our data indicate that microbial communities in sandy sediments digest and transform labile parts of photosynthesis-derived particulate organic matter and likely release more stable, glucose-depleted residual glycans of unknown structures, quantities, and residence times into the ocean, thus modulating the glycan composition of marine coastal waters.

## Introduction

Continental shelf ecosystems contribute 15%–21% of global primary production [1] of which up to 50% reaches the shallow seafloor. About half of the continental shelf area is covered by sandy sediments [2, 3]. Their high permeability enhances the advective flow of bottom water with organic matter (OM) [4, 5]. Heterotrophic benthic bacteria remineralize this imported OM as well as OM derived from benthic primary production [2, 4, 6].

A major fraction of the OM consists of polysaccharides that phytoplankton produces for energy storage, for cell wall building blocks or as exudates. Glycans constitute up to 80% of the algae dry weight, depending on the species and growth phase [7]. They are structurally complex, in terms of linkage, configuration, and diversity of monosaccharide building blocks [8]. This structural diversity makes marine glycans an important theme of ongoing research; reviewed in, e.g. [9]. For the utilization of these carbohydrates, heterotrophic bacteria use a diverse set of carbohydrate-active enzymes (CAZymes). They include glycoside hydrolases (GH), polysaccharide lyases (PL), carbohydrate esterases (CE), and accessory proteins, such as proteins carrying carbohydrate-binding modules; CBMs [10]. The number of CAZymes required for the degradation of a glycan scales linearly with its structural complexity [11]. For example, for the digestion of complex, branched, and highly sulfated fucoidan consisting of multiple monosaccharides, including methylpentose (fucose) from brown algae, many enzymes are required [12]. By contrast, for the degradation of laminarin, which is the most abundant marine glycan and contributes 26% ± 17% to the particulate organic carbon pool [13], two or three enzymes are—at least *ex situ*—sufficient to degrade laminarin into glucose [14]. Due to the structural diversity, direct quantification of specific polysaccharides in the environment remains technologically challenging [15]. Inventories of bacterial CAZymes, therefore, offer an alternative approach for studying bacterial glycan utilization [16, 17].

While the bacterial glycan degradation in temperate surface waters was shown to be highly dynamic, e.g. [17, 18], benthic bacterial communities have limited seasonality [19, 20]. Polar regions with their prolonged periods of complete darkness in winter and 24 h of sunlight in spring and summer are ideal environments to study the seasonality of bacterial glycan degradation. Arctic fjords of Svalbard (74–81°N) have strong peaks of primary production in spring and summer, fueling the entire coastal ecosystem, including its sediments [21]. Of particular importance is benthic photosynthesis, which adds a significant amount of fresh, labile OM to the seafloor in shallow coastal regions. For example, in Kongsfjorden, microphytobenthos primary production is comparable to those from pelagic production [22]. In addition, there is a terrestrial input from the glacial run-off and a contribution of ice algae, which are also both sources of OM, which vary with season. Large datasets from Svalbard fjords have repeatedly underscored seasonal changes in respiration, sulfate reduction, and mineralization in these permanently cold sediments (−1°C to +4°C); for review, see [23]. Respiration is mostly driven by the input of fresh OM and it is plausible to assume that fresh OM will also drive seasonal succession of heterotrophic bacteria in sandy surface sediments. However, benthic and pelagic microbial communities differ fundamentally in their ability to access high molecular weight substrates such as polysaccharides in arctic sediments [24]. Furthermore, rRNA gene-based studies showed a stable community composition over 2 years in coastal sands from Isfjorden [19]. Since studies based on the comparative sequence analysis of rRNA genes are limited in taxonomic resolution, we revisited Isfjorden sediments and studied them by a combination of metagenomics and metatranscriptomics. Thereby, we expected to detect subtle differences in the gene repertoire of bacteria and in gene expression. In this study, we tested the following three hypotheses: (i) metagenomic (and metatranscriptomics) data reveal seasonal differences in the genes encoding glycan utilization. Furthermore, benthic bacterial communities respond to the seasonally changing input of fresh OM by changing the regulation of genes encoding CAZymes. (ii) The utilization of continuously available, less labile substrates explains the high overall stability in the benthic bacterial community composition. (iii) The main glycans used by heterotrophic benthic bacteria change between seasons and can be predicted based on gene expressions. For addressing the third hypothesis, we additionally performed glycan analyses.

**Figure 1:**
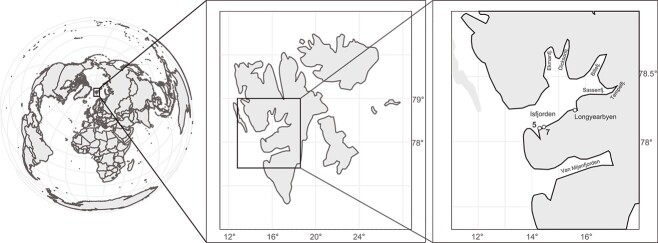
Sampling area in Isfjorden, Svalbard; surface sediments were retrieved from two shallow sites (78°N, 4–8-m water depth) close to Kapp Dresselhuys.

## Materials and methods

### Sampling

Sediment samples (fine sand) were taken from Isfjorden, Svalbard (Fig. 1), using a van Veen grab. Surface layers (0–2 cm depth) were sampled at Station 5 (78.11°N/14.35°E) and at Station 7 (78.10°N/14.38°E) in 2017 (20 December), 2018 (6 February, 1 May, and 17 December), and 2019 (25 April). Sediment temperatures ranged between −0.6°C and 2.2°C, water depth was between 2.7 and 8.8 m. Chlorophyll *a* in seawater was 6.3–8 μg l^−1^ in spring and 0.4 μg l^−1^ in winter. In sediments, Chlorophyll *a* was 0.4–1.0 μg ml^−1^ in spring and was 0.2–0.3 μg ml^−1^ in winter. Contextual data have been reported previously [19]. In addition, sediments, porewater, and bottom water (sampled above the sediment surface; hereafter, referred to as “overlying seawater,” OSW) were sampled for glycan analysis using a Shipek-type grab at Station 5 in 2022 (29 April and 2 May). All samples were immediately frozen in dry ice.

### DNA and RNA extraction

DNA for metagenome analysis was extracted from the 0–2-cm depth horizon of selected sediment samples of December 2017, February 2018, May 2018, December 2018, and April 2019 after Zhou *et al*. [25], including three additional freeze-thawing steps. RNA was extracted from December 2017, February 2018, and May 2018 samples using RNeasy PowerSoil Total RNA Kit (QIAGEN, Hilden, Germany) according to the manufacturer’s recommendation with minor modifications. For an overview of samples and details, see Supplementary Table S1 and Supplementary Information.

### Library preparation, sequencing, assembly, and binning

Illumina-compatible libraries were prepared from genomic DNA with NEBNext Ultra™ DNA v2 Library Prep Kit for Illumina (New England Biolabs, Frankfurt, Germany), starting with initial DNA fragmentation using a Covaris S2 ultrasonicator (Covaris, Woburn, MA, USA). Illumina-compatible RNAseq libraries were prepared from total RNA with NEBNext UltraII Directional RNA Library Prep Kit for Illumina (New England Biolabs). In addition, three Illumina-compatible RNAseq libraries from Station 5 were prepared from bacterial rRNA-depleted RNA using the Illumina Ribo-Zero rRNA depletion kit (“Bacteria”). Metagenome and metatranscriptome sequencing were done on a HiSeq 2500 System (Illumina, San Diego, CA, USA, 2 × 250 bases) at the Max Planck-Genome Center in Cologne (Germany). Detailed settings of the programs used for sequence analysis are given in the Supplementary Information. In short, sequences were quality-controlled using BBTools v37.62 (quality < 20, minimum length 140 nt). Coverage of sequence diversity was analyzed using nonpareil v3.303 [26]. Assembly of reads was done with SPAdes v3.13.1 [27] (meta option) and the quality was evaluated using QUAST v4.5 [28]. Contigs < 1 kb length were excluded from further analyses.

For each dataset, binning was done using MaxBin v2.2.7 [29] and MetaBAT v2:2.15 [30]. Bin refinement was performed using DAS_Tool v1.1.2 [31]. Mapping for differential coverage binning was done using bbmap v38.70 [32] at default settings and a minid = 0.99. Dereplication was performed with dRep v3.1.1 [33] (−comp 50, −con 15) and classification using the Genome Taxonomy Database (GTDB)-Tk v2.1.1 and the GTDB release r214 [34]. Completeness and contamination were assessed in checkM v1.0.7 [35].

### Gene annotation and analyses

Gene predictions and annotations from bins were done using Prokka v1.14.6 [36], dbCAN (run_dbCAN v2.0.11 workflow; https://github.com/linnabrown/run_dbcan) [37], Swiss-Prot release 2021_04 [38], SulfAtlas v1.0 [39], and transporterDB (download Oct 2021) [40]. The latter three databases were searched using DIAMOND blastp (v2.0.15.153) [41]. Results were filtered for the best hit using the enveomics script BlastTab.best_hit_sorted [42] (>60% identity, query coverage > 70%).

CAZyme annotations obtained from dbCAN were accepted when two of the three integrated annotation methods (HMMER v3.3.2, diamond v2.0.9.147, Hotpep version included in run_dbCAN workflow) matched [37].

### Transcriptomic analyses

Quality-controlled RNA reads were sorted using SortMeRNA 4.0.4 [43]. Reads identified as rRNA were taxonomically classified by using the SILVAngs pipeline (https://ngs.arb-silva.de/silvangs/, release 138.1) [44]. All reads that were not classified as rRNA or tRNA were considered as mRNA.

Annotation of transcripts was done by mapping mRNA to predicted genes from metagenomics contigs and bins using DIAMOND blastx (v2.0.15.153) [41]. Results were filtered for the best hit using the enveomics script BlastTab.best_hit_sorted [42] (>60% identity, query coverage > 70%). Values of transcripts per million (TPM) mapped reads were calculated after normalization by gene length.

Calculations of predicted monosaccharide patterns were done based on the expression of GH genes (TPM) by referring enzyme activities given in the CAZy database to one or several monosaccharide types released/degraded. Details are provided in the Supplementary Information; the script was deposited on Gitlab (https://gitlab.mpi-bremen.de/smiksch/gh_family_to_monosaccharides). Data transformation and plotting were done using R and the tidyverse packages [45].

### Monosaccharide analysis

Polysaccharides extracted from sediment, porewater, and OSW were acid hydrolyzed and the resulting monosaccharides were analyzed using high-performance anion exchange chromatography (HPAEC) with pulsed amperometric detection (PAD) according to Vidal-Melgosa *et al*. [46]. For details, see Supplementary Information. Values measured for calibration standards having high monosaccharide concentrations were consistent between injections during the chromatographic run. Low-concentrated calibration standards, however, showed much lower values at the second injection. To account for this decrease in detector sensitivity with time, a threshold concentration for each monosaccharide was set to the value at which the variation between two injections was ±20%. Values lower than the threshold concentrations defined for each monosaccharide were rejected.

## Results

### Bacterial community composition as revealed by rRNA read frequencies

**Figure 2:**
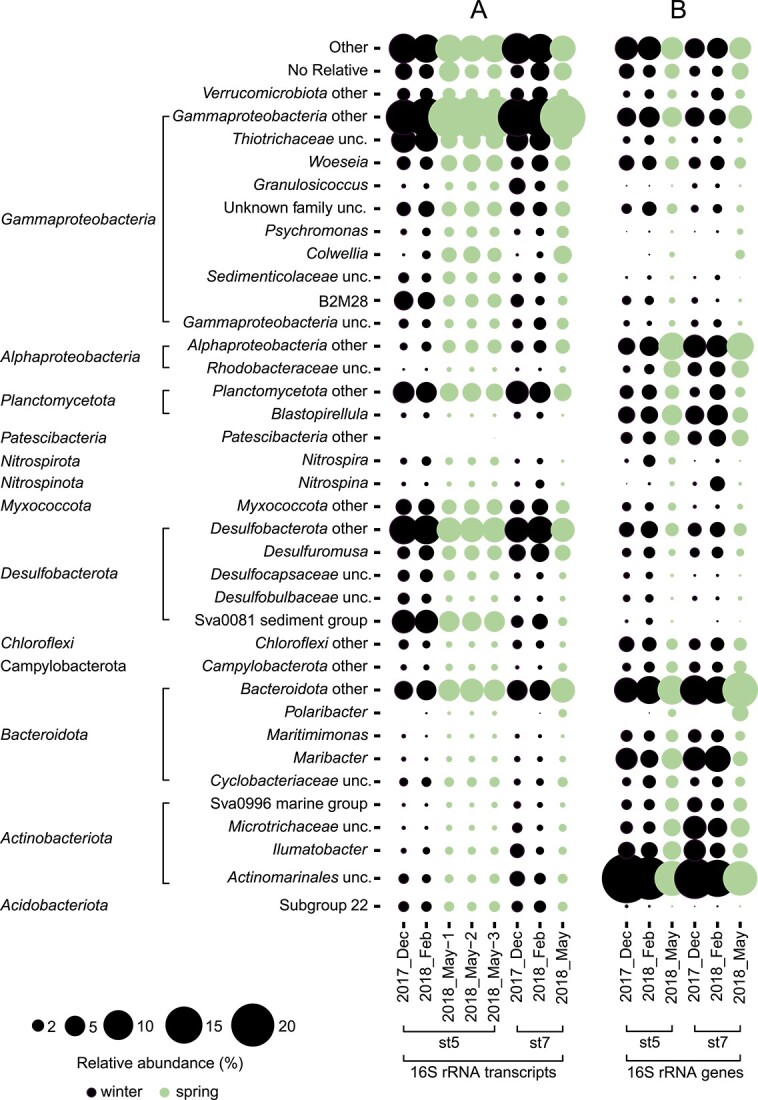
Major taxa of bacterial communities in Svalbard surface sediments are seasonally stable; comparison of the benthic community composition as revealed by (A) rRNA read frequencies in metatranscriptomes and (B) 16S rRNA genes frequencies from amplicon tag sequencing; extracted from [19]; for classification, 20 000 reads were randomly subsampled from each dataset and were submitted to SILVAngs (https://ngs.arb-silva.de/silvangs/, release 138.1) [44]; only taxa with a read frequency of >2% are shown, minor taxa are summarized as “other”; both rRNA and rRNA genes revealed a stable bacterial community in winter and spring; only minor taxa such as *Colwellia* and *Polaribacter* spp. showed a clear seasonal variation in abundance.

As a proxy for activity of a population, we used rRNA read frequencies from 11 metatranscriptomes recovered from Isfjorden sediments (December 2017, February 2018, May 2018; Fig. 2A). The rRNA read frequencies of the majority of clades were not remarkably different between seasons. Notable exceptions were rRNA reads affiliated with the genera *Colwellia* and *Polaribacter* that showed increased relative abundance from winter (average of 0.6% and 0.1% of total 16S rRNA reads, respectively) to spring (average of 3.3% and 0.6%, respectively).

Although no clear differences in the community composition between seasons were detected, relative abundance in metatranscriptomic 16S rRNA versus amplicon 16S rRNA genes [19] differed for several taxa (Fig. 2B). A greater relative abundance in metatranscriptomic 16S rRNA versus amplicon 16S rRNA genes was determined for *Verrucomicrobiota* (2.3 ± 0.4% rRNA vs. 1.3 ± 0.5% amplicon rRNA genes), *Planctomycetota* (4.8 ± 0.9% vs. 2.4 ± 0.7%), *Desulfobacterota* (7.9 ± 1.1% vs. 2.8 ± 0.5%), *Thiotrichaceae* (5.0 ± 1.3% vs. 0.9 ± 0.3.), and *Myxococcota* (3.0 ± 0.3% vs. 0.8 ± 0.4%). By contrast, a lower relative abundance in rRNA read frequencies in metatranscriptomic 16S rRNA versus amplicon 16S rRNA genes was found for *Blastopirellula* (0.6 ± 0.2% vs. 4.2 ± 0.8%), *Bacteroidota* (*Maribacter* 0.6 ± 0.1% vs. 5.4 ± 1.8%; *Maritimimonas* 0.4 ± 0.1 vs. 2.0 ± 0.4%), and *Actinomarinales* unc. (1.6 ± 0.6% vs. 17.8 ± 5.3%). The metatranscriptomes comprised only few archaeal 16S rRNA sequences (<1%).

### Addressing changes in functional potential of benthic bacteria by omics

Metagenomes and metatranscriptomes from Svalbard sediments were used to study possible changes in the functional potential of the bacterial community and to detect differences in the genomic repertoire between species of the same genus and in gene expression of CAZymes. Three metagenomes were obtained from Station 5 samples in winter (December 2017, February 2018, December 2018; hereafter, referred to as “winter”) and three metagenomes in spring (Station 5: May 2018, April 2019; Station 7: April 2019; hereafter, referred to as “spring”). Nonpareil, a redundancy-based approach to assess the level of coverage, ranged between 0.46 and 0.5 for all metagenomes, indicating that about half of the total diversity was covered (Supplementary Table S2). A total of 9 207 104 genes were predicted of which about one-third remained hypotheticals after annotation. A total of 183 bins (16–42 bins per sample) were recovered of which 36 bins (Supplementary Table S3) were selected based on the diversity and quality for further analysis. The bins represented all major taxa previously found in sandy surface sediments [19], including *Acidimicrobiia*, *Bacteroidia*, *Desulfobacteria*, *Planctomycetota*, and *Gammaproteobacteria*.

### Seasonal expression of bins

**Figure 3:**
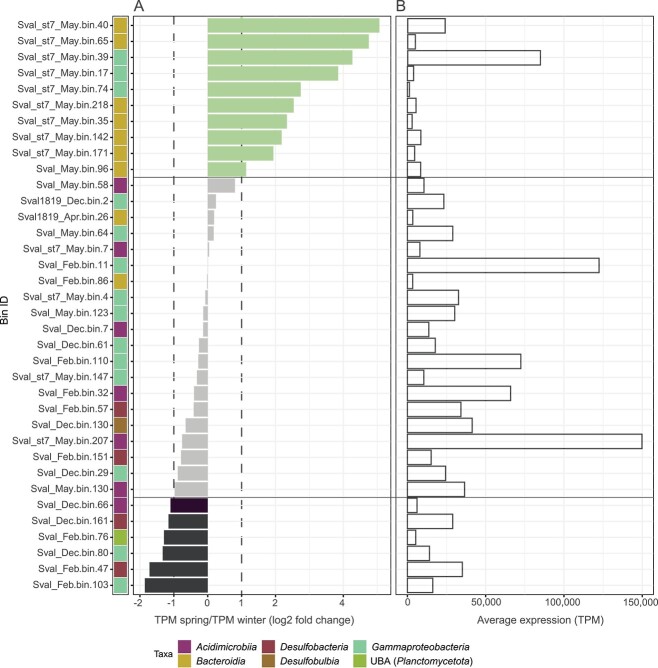
Expression of bins from Svalbard sediment metagenomes; (A) changes of bin expression given as a ratio of TPM mapped reads from spring metatranscriptomes divided by TPM mapped reads from winter metatranscriptomes; values are plotted as log2-fold change; a bin was defined being upregulated in spring for log2-fold changes of >1 (corresponding to a ratio TPM spring/TPM winter of >2; green bars) and being upregulated in winter for log2-fold changes of <−1 (corresponding to a ratio TPM spring/TPM winter of <0.5; black bars); gray bars show less regulated bins not matching these thresholds; (B) expression of bins in TPM given as an average of all sampling time points and metatranscriptomes; some bins of *Bacteroidia *and *Gammaproteobacteria *were upregulated on mRNA level in spring, while clade UBA9214 (Bins 80 and 103), *Acidimicrobiia *(Bin 66), and *Desulfobacteria* bins (Bins 47 and 161) were more expressed in winter; most highly expressed Bins 207 and 11 were less regulated.

As a proxy for activity, 11 metatranscriptomes (December 2017, February 2018, May 2018) were mapped on the 36 bins. A bin was considered being upregulated in spring when the ratio (average spring TPM mapped reads/average winter TPM mapped reads) was ≥2 (=log2-fold change of >1; green bars, Fig. 3A) and being upregulated in winter when the ratio (average spring TPM mapped reads/average winter TPM mapped reads) was <0.5 (=log2-fold change of <−1; black bars). Bins with log2-fold changes −1 ≤ *x* ≤ 1 were considered as constantly expressed and therefore unregulated (gray bars). According to this definition, 10 of 36 bins were upregulated in spring. Of this group, seven belonged to *Bacteroidia* and three belonged to *Gammaproteobacteria*. Among the most upregulated bins were *Flavobacteraceae*-bin Sval_st7_May.bin.40 and *Colwellia*-bin Sval_st7_May.bin.39 with a 33-fold and 19-fold higher TPM in spring versus winter, respectively. In winter, six bins showed an increased expression (Fig. 3A, black bars) affiliated with *Gammaproteobacteria*, *Desulfobacteria*, *Acidimicrobiia*, and *Planctomycetota*. The majority of unregulated bins (9 of 20 bins, Fig. 3A, gray bars) were *Gammaproteobacteria*. Many of these bins had high TPM values (Fig. 3B).

### Carbohydrate-active enzymes and polysaccharide utilization loci

We focused our analysis on CAZymes, in particular on GH families, as they can be used as a proxy for polysaccharide degradation. Overall, GH23 (peptidoglycan lyase) was the most abundant family in the metagenomes (Supplementary Fig. S1) and was not remarkably changing between seasons. Most members of the GH23 family have peptidoglycan lyase activity and are widely distributed among many phyla such as *Proteobacteria* and *Firmicutes* [10]. In winter metagenomes, e.g. GH29 (fucosidase), GH106 (rhamnosidase), and GH165 (galactosidase) were more abundant than in summer with a log2-fold increase of −1.2 to −2.2, yet with lower frequencies than the less regulated representatives GH16, GH17, GH23, and GH103. Spring-induced GH were less frequent (below threshold of >0.1% metagenomics abundance).

The number of GH in the bins varied between 3 and 16 GH Mbp^−1^ (Supplementary Fig. S2, Supplementary Table S4) and the number of total CAZymes (GH, CE, and PL) varied between 5 and 22 Mbp^−1^. Three of the bins showed a high density of peptidases with 8–11 Mbp^−1^ but comprised only 6–9 CAZymes Mbp^−1^. Major substrates expected to be consumed by these *Bacteroidia* were laminarin or other β-glucans (GH16_3, GH2, GH3, GH149, GH17, and GH30_1), α-glucans such as glycogen (GH13, GH13_19, GH31, and GH65), mannans (GH92), xylans (GH3), and alginates (PL7 and PL17).

Polysaccharide utilization loci (PUL) are structured genomic regions that are used to predict the substrate of heterotrophic bacteria and are common in *Bacteroidia* [17, 47]. Canonical bacteroidetal PUL include a pair of susCD-like transporter genes and ≥2 CAZyme genes, like GH, PL, CE, or CBM, within a 10-genes-sliding window [48]. Automated prediction of canonical PUL and PUL-like structures (defined as *susCD* pair or a single *susC* and ≥ 1 CAZyme) identified 16 loci (Supplementary Table S4) in the 7 Bacteroidia bins that were upregulated in spring. Another 11 loci were identified with multiple CAZymes, but no *susCD.* The two seasonally unregulated bins, Sval1819_Apr.bin.26 and Sval_Feb.bin.86 (Fig. 3, gray bars) did not comprise contigs with canonical PUL or PUL-like structures, but four and eight single CAZymes Mbp^−1^ (Supplementary Fig. S2).

### Seasonal changes in gene expression

To analyze the changes in gene expression, the average relative frequency of transcripts was calculated for winter and spring (Supplementary Fig. S3, Supplementary Table S5). The 10 most expressed genes comprised only hypothetical proteins. Expressions of most of these unknowns did not differ between seasons, but those contribute evenly to the gene expression by the sediment community. Genes related to photosynthesis were highly upregulated in spring: besides photosystem I- and II-related genes, other genes of presumably photosynthetic organisms, e.g. ribulose bisphosphate carboxylase, had up to 14-fold higher TPM values in spring than in winter. Furthermore, ammonia channel proteins/transporters and cytochromes were also upregulated in spring. By contrast, genes involved in nitrogen and sulfur cycling were upregulated in winter (Supplementary Fig. S4). These are, in particular genes for respiration, e.g. nitrite reductase and nitrate reductase (1.5 and 1.3 log2-fold change TPM winter vs. spring, respectively), as well as dissimilatory sulfate reductase (log2-fold change TPM winter vs. spring of 1.0) and adenylylsufate reductase (log2-fold change TPM winter vs. spring of 1.1).

**Figure 4:**
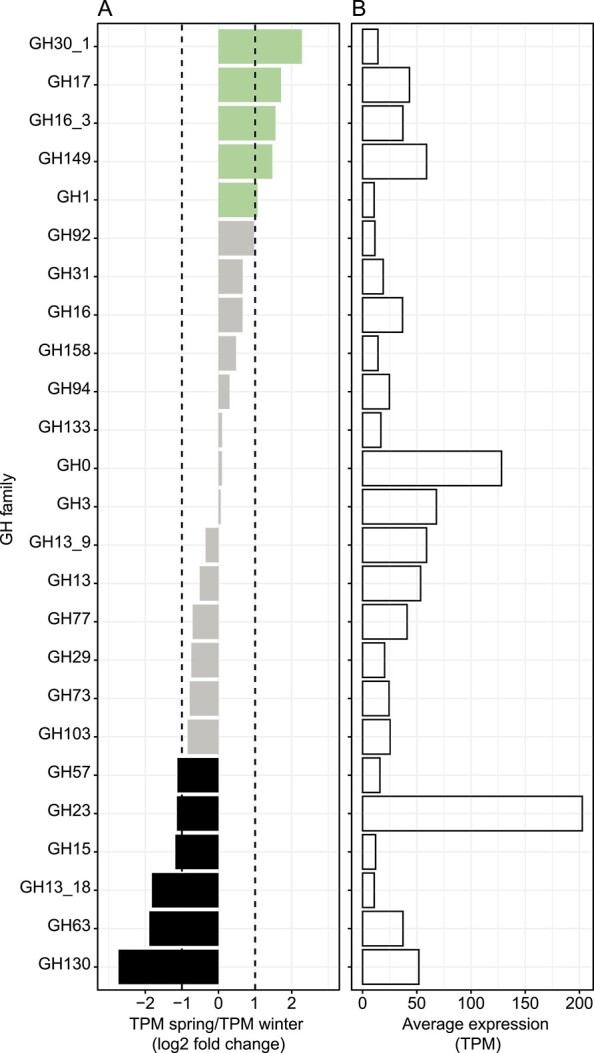
Expression of GH families in spring and winter; β-glucan utilization is upregulated in spring (GH17, GH16_3, and GH149), while α-glucan utilization is more prominent in winter (GH63, GH15, and GH57); (A) changes of GH expression are given as a ratio of TPM in spring metatranscriptomes divided by TPM in winter metatranscriptomes; values are plotted as log2-fold change; a GH family was defined as being upregulated in spring when log2-fold changes were >1 (TPM spring/TPM winter >2, green bars) and being upregulated in winter when log2-fold changes were <−1 (TPM spring/TPM winter < 0.5, black bars); gray bars show unregulated GH families not matching these thresholds; (B) average expression of GH families in TPM calculated from metatranscriptomes from all sampling time points; GH families shown are expressed in spring and winter (no infinite fold change) with TPM values > 10.

Most prominent GH families upregulated in spring were GH30_1, GH17, GH16_3, and GH149 (Fig. 4). Enzymes of these families comprised β-glucanases and are likely degrading laminarin. GH149 also acts on β-1,3-linked glucan as phosphorylase. GH families that were downregulated in spring included GH130, GH63, GH13_18, GH15, GH23, and GH57. Enzymes characterized within these families showed a diverse range of activities such as mannoside phosphorylases (GH130), α-glucosidases (GH63), α-glycoside phosphorylases (GH13_18), glucan-1,4-α-glucosidase (e.g. glucoamylase, trehalase; GH15), peptidoglycan lyases (GH23), and α-glucanases (GH57).

### Monosaccharide concentrations in sediments

**Figure 5:**
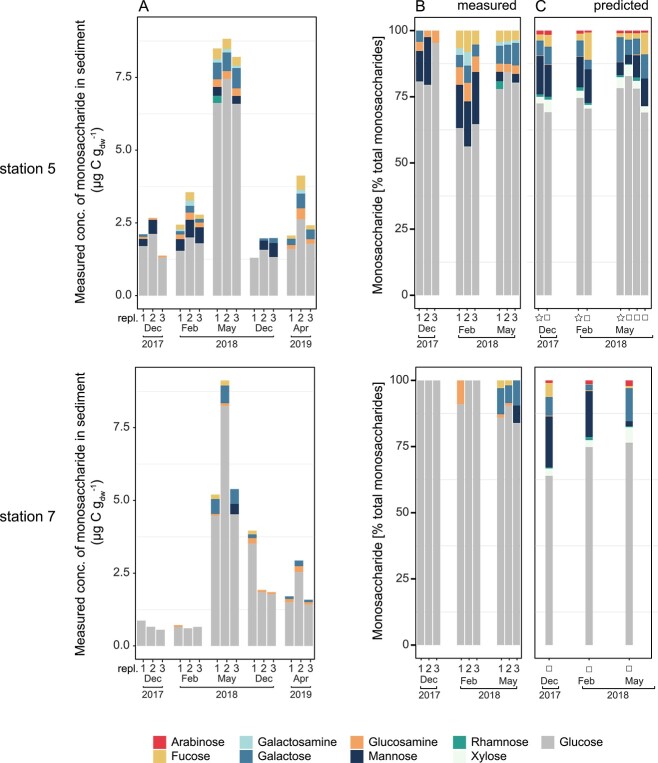
Monosaccharide concentrations measured and predicted based on expression patterns of GH genes in Svalbard sediments; glycans from the water extracts of sediment samples were acid hydrolyzed and the resulting monosaccharides were measured by HPAEC-PAD analysis; (A) concentrations and (B) relative fractions of total measured monosaccharides; (C) monosaccharide utilization deduced from predicted functions of expression patterns of GH genes; star, data from rRNA-depleted metatranscriptome; square, data from “full” metatranscriptome; all samples were dominated by glucose; in particular, in spring 2018, glucose and galactose concentrations strongly increased, while mannose was more prominent in winter samples; the measured monosaccharide composition is in line with the predicted trends of monosaccharide utilization patterns based on GHs’ expression.

To link the gene expression of CAZymes with glycan concentrations in the sediment, we extracted glycans from the sediment with MilliQ water and quantified their monosaccharides after acid hydrolysis. The monosaccharide composition of the water extracts was dominated by glucose, accounting for 50%–80% of total monosaccharides (Fig. 5). In spring 2019, total monosaccharide content (sum of concentrations of all different monosaccharides) was on average lower than in spring 2018 (Station 5: ~3 μg C gdw^−1^ sediment vs. ~8.5 μg C gdw^−1^ and Station 7: 2.5 vs. 7.5 μg C gdw^−1^ in 2019 and 2018, respectively). Samples from station 7 (December 2017 and February 2018) contained only glucose in measurable amounts, while other monosaccharides were below the detection limit. Other abundant monosaccharides in our samples were mannose and galactose. In winter, the concentration of mannose increased by a factor of ~2.2 from 0.08 μg C gdw^−1^ sediment in spring to 0.17 μg C gdw^−1^ (average for station 5 and 7). By contrast, spring samples had a 7.9-fold higher concentration of galactose (winter: 0.05 μg C gdw^−1^; spring: 0.41 μg C gdw^−1^ sediment) and a 4.3-fold higher concentration of fucose than in winter (winter: 0.04 μg C gdw^−1^; spring: 0.17 μg C gdw^−1^ sediment).

In an additional sampling campaign at Station 5 in April 2022, we collected porewater and OSW along with sediment samples. Total concentrations of monosaccharides were similar to those measured in spring 2019 with on average 2.9 ± 2.6 μg C gdw^−1^ sediment for four replicate grabs (data not shown). Total concentrations in porewater were high, with 1107 ± 484 μg C l^−1^ being 18-fold higher than those measured for OSW (62 ± 101 μg C l^−1^, Supplementary Fig. S5, Supplementary Table S6). The monosaccharide composition differed between sediments and porewater: the porewater monosaccharide spectrum was not dominated by glucose, but it mostly had an even contribution of glucose, arabinose, fucose, galactose, glucosamine, and xylose (Supplementary Table S6).

### Predicting monosaccharide utilization based on GH expression data

The frequency of mRNA reads annotated as GH was used to predict monosaccharide utilization in Svalbard sediments and to test if the predicted patterns correlate with the measured monosaccharide concentrations. We assigned one or more monosaccharides to each detected GH family based on information given in the CAZy database (matrix available as Supplementary Table S7). The monosaccharide utilization pattern predicted based on the transcriptomic data (Fig. 5C) was similar to the pattern of measured monosaccharides at Station 5 (Fig. 5B): it indicated a dominance of glucose utilization, accounting for >60% of the total used monosaccharides in sediments in spring. Analog to measured monosaccharide concentrations, transcripts mapping to mannose-related GH families were more prominent in winter, while galactose-related GH families were more abundant in spring predictions. In line with monosaccharide measurements, fucose, rhamnose, arabinose, and xylose utilizations were detected, though in a less seasonally consistent manner.

Concentrations of most monosaccharides were below detection threshold at Station 7; thus, a comparison of measured and predicted monosaccharide composition is not meaningful.

## Discussion

Bacterial communities in temperate and polar sediments were reported to be seasonally stable based on 16S rRNA gene frequencies [19]. In this study, we showed that also the ribosomal RNA expression of most taxa did not remarkably change between winter and spring (Fig. 2), supporting previous findings. Although the rRNA concentrations of diverse natural bacterial communities cannot be metrically linked to real-time activities due to the differences in life histories, life strategies, and nongrowth activities [49], rRNA frequencies have been used as a proxy for growth potential and activity of a population due to the relationship between cellular ribosome content and the ability to synthesize proteins [50]. Our data show that abundant taxa (>5% of total rRNA reads) such as *Actinobacteria*, *Bacteroidia* (except *Polaribacter)*, *Desulfobacterota*, *Myxococcota*, and *Woesiaceae*, were seasonally stable. The detected stability gives a first hint that a major part of the bacterial community is thriving on constantly available substrates rather than seasonally fluctuating substrates like laminarin.

### *Colwellia* and *Polaribacter* are prominent in spring

Despite the stability of major phyla, two genera, *Polaribacter* (*Bacteroidia*) and *Colwellia* (*Gammaproteobacteria*) showed a strong increase in spring, with average relative abundances rising from <0.1% to 1.1% and from 0.3% to 4.1% of total rRNA reads, respectively. Both genera are known for the degradation of various algal polysaccharides and are tightly associated with phytoplankton blooms; e.g. [16, 18, 51-53]. In particular, members of the genus *Colwellia* have been reported to be seasonally abundant in Arctic and Antarctic waters and sea ice [53-55]. The increase of *Colwellia* rRNA frequency in spring versus winter went along with a 19- to 36-fold higher expression of mRNA that mapped on the *Colwellia* bin Sval_st7_May.bin.39 (Fig. 3). Two contigs contained PUL-like loci (no *susCD*; Supplementary Fig. S6). Of the genes in these two loci, all GH and CBM genes were expressed in the spring metatranscriptomes, while they were not detected in winter metatranscriptomes. In particular, GH17, GH16_3, and GH149, indicative of the degradation of laminarin as the main storage glycan of diatoms [13] and brown algae were upregulated. The most likely explanation for their absence in our winter metatranscriptomes is a low expression combined with insufficient depth of sequencing. Only GH73 and GH103 (both genes outside the PUL-like loci) were slightly expressed in winter metatranscriptomes (TPM ~ 0.2). GH73 (peptidoglycan hydrolases) was higher expressed in winter than in spring, which is reasonable as the recycling of bacterial cell compounds becomes relatively more important. This *Colwellia* bin with a genome size of 3.65 Mbp (95% completeness; 1.9% contamination) represents a novel species according to the ANI-based genomic similarity criteria for delineating species used by the GTDB [56]. Its closest phylogenetic relative is an uncultured *Colwellia* sp. from marine water (ANI 85.9%; bioproject PRJEB37807, BioSample SAMEA9694887) with a genome size of 4.9 Mbp. We conclude that the high abundance and pronounced seasonality of *Colwellia* ask for future studies on the ecology of this gammaproteobacterial genus in polar systems.

Besides *Colwellia*, seven *Bacteroidia* bins showed a strong upregulation in spring (Fig. 3). These bins are on average of a size of 2.86 ± 0.35 Mbps and thereby significantly larger than the 2.0 Mbp bins of *Bacteroidia* obtained from temperate surface waters [17]. Surface water Bacteroidia are characterized by a wealth of PUL that encode CAZymes, carbohydrate-binding proteins, and SusCD-like transporters; for review, see [57]. Like planktonic *Bacteroidia*, the benthic bins also affiliated with the family *Flavobacteriaceae* and show a similar genomic organization regarding polysaccharide degradation. The variability of GH in our *Bacteroidia* bins was high (3–16 GH Mbp^−1^; Supplementary Fig. S2) and was only slightly lower than that found for pelagic *Polaribacter* spp.; e.g. [16-18]. Ratios of annotated degradative CAZymes versus peptidases suggest a niche separation of *Bacteroidia* into carbohydrate (four/seven bins) and protein degradation (three/seven bins; Supplementary Fig. S2).

### *Desulfobacteria* is an abundant taxon slightly elevated in winter

Bins of sulfate-reducing *Desulfobacteria* (Fig. 3) showed an elevated relative expression in winter. The same was observed for key genes involved in sulfur cycling (Supplementary Fig. S4), both indicating a more prominent role of sulfur cycling in winter samples, potentially linked to more anoxia in the absence of benthic photosynthesis. Most sulfate-reducing bacteria rely on low molecular products, such as fatty acids and hydrogen [58], which are available throughout the year, while fresh, complex organic material gets limited in winter. Canonical denitrification as indicated by genes for nitrate and nitrite reductases (*nir* and *nar*) was more prominent in winter (Supplementary Fig. S4), which supported extended phases of anoxia.

### Relative utilization of β-glucans increases in spring

In spring, the use of algae-derived β-glucans was most prominent by an elevated expression of mRNA of GH families GH30_1, GH17, GH16_3, and GH149, together indicating an increased degradation of laminarin (Fig. 4). Several GH families with galactosidase activities (according to CAZy database) [10] were also upregulated in spring such as GH1, GH4, or GH42 (Supplementary Table S5). Galactose has been described to be a main building block of several marine algal polysaccharides, like agar and carrageenan, which are important components of macroalgae cell walls [59]. Hydrolysis of such labile algal polysaccharides would be plausible to be induced in spring when algal biomass is increasing. The importance of laminarin as carbon source for benthic microbes is supported by short-term incubations of intact sediment cores from different fjords at Svalbard with fluorescently labeled polysaccharides, which showed a rapid hydrolysis of laminarin in surface sediments [24]. Sources of laminarin are micro- and macroalgae that extensively colonize coastal habitats in Arctic fjords such as Isfjorden and Kongsfjorden [60-62]. While Arctic kelp can grow even during polar night using stored carbohydrates and sugar alcohols derived from summer/autumn photosynthetic periods [63, 64], microalgae such as diatoms show a strong seasonality [60]. At the time point of spring sampling, chlorophyll *a* concentrations in seawater and sediments, as well as 16S rRNA amplicon frequencies from chloroplasts, were clearly higher than in winter [19], supporting the presence of a current or recent phytoplankton bloom.

### Relative utilization of α-glucans increases in winter

Transcript levels for the degradation of α-glucans like glycogen (GH63, GH15, and GH57) increased in winter (between log2-fold change −1.1 and −1.9) but were detected in spring, too. α-Glucans are the intracellular storage products of not only many heterotrophic bacteria [65], but they are also the intracellular storage products of animals and protists as well as some fungi [66, 67]. Therefore, glycogen is continuously available, either in intracellular pools or recycled from bacterial and animal biomass. We assume that, in spring, benthic bacteria use large amounts of available glycans and transform part of them into glycogen, thus making it available later during the year. Thus, unlike laminarin, glycogen is a constantly available carbon source contributing to the high stability in the bacterial community composition. GH23 transcripts were also upregulated in winter. They likely encode hydrolysis of peptidoglycan [68]. This could be a result of starvation since bacteria are known to reduce their size and use cell wall compounds as energy source [69].

### Utilization of constantly available substrates

Besides glycogen, we found multiple indications that other substrates are continuously used, likely contributing to the high stability of benthic bacterial communities. The transcription of most of the abundant GH families was independent of polar day and night and was not remarkably regulated across seasons (Fig. 4). Among these GH families, there were mannosidases (GH92), α-glucanases (GH31, GH133, GH13_9, and GH77), β-glucanases (GH16, GH158), fucosidases (GH29), peptidoglycan lyases (GH73 and GH103), and families without a clear substrate affiliation (GH94, GH0, GH3, and GH13).

Further constant carbon sources are chitin and mucin, which are both mostly of animal origin. Benthic meiofaunal and macrofaunal density and diversity in the close by Kongsfjorden have been shown to be stable throughout the year [70]. Chitin is the most abundant polysaccharide in surface marine sediments [71], yet we could not identify high transcription levels of known chitinases; e.g. GH18 [72], in our samples. However, in the Bins Sval_st7_May.bin.207 and Sval_Feb_bin.32, we did find expressed chitosan disaccharide transporters as well as key genes for further breakdown catalyzing deacetylation (chitooligosaccharide deacetylase) and hexosaminidases (GH3). Both bins were classified as *Acidimicrobiia* and were highly expressed in winter and spring (Fig. 3).

Mucins are glycoproteins copiously secreted by marine fauna, in particular by invertebrates [73]. They constitute a complex class of energy-rich substrates containing a protein backbone with side chains of oligosaccharides, which can be very diverse in nature, covering glycans composed of different monosaccharide building blocks [74]. Mucus is a potent substrate for marine microbes. Hannides and colleagues [75] showed a strong priming effect of gastropod mucus on benthic OM remineralization. Key enzymes for mucin degradation have been identified for gut bacteria comprising sialidases (GH33), fucosidases (GH29 and GH95), *N*-acetylgalactosaminidases (GH101 and 129), *N* -acetylglucosaminidases (GH84 and GH85), galactosidases (GH2, GH20, and GH42), and proteases [76, 77]. These were all present in our metagenomes and were expressed either all year or preferentially in winter metatranscriptomes. This corroborates that mucins are important substrates for benthic bacteria.

### Monosaccharide measurements are consistent with carbohydrate-active enzyme expression

Glucose concentrations were seasonal with clear maxima in spring. Their up to 4-fold increase was consistent with higher transcription levels of β-1,3-glucan degradation genes, indicating substrate-related induction. Also in winter, glucose remained an important substrate, likely because the α-glucan storage products of animals and bacteria were recycled. Another direct relationship between the abundance of monosaccharides and transcript frequency of degradative enzymes was observed for mannose-containing substrates whose concentrations were higher in winter (0.38 ± 0.16 μg C g^−1^ sediment) compared to spring (0.16 ± 0.10 μg C g^−1^ sediment). Correspondingly, genes belonging to GH130 family (including activities like β-1,4-mannosylglucose phosphorylase, β-1,4-mannooligosaccharide phosphorylase, and β-1,4-mannosyl-*N*-acetyl-glucosamine phosphorylase) as well as genes encoding GH63 (α-glucosidase and α-mannosidase) and GH113 (β-mannanase and β-mannosidase) were upregulated toward winter. The α- and β-mannans are known to be important compounds of diatom cell walls [78, 79]. These cell walls are considered to be semi-labile OM and therefore are relatively more important in winter when labile glucans such as laminarin are long gone.

Overall, this study suggests that the transcription frequency of GH families is linked to monosaccharide concentrations in the natural environment. This comparison between detected monosaccharides and expressed GH should ideally be extended to the glycan level, so types and substrate classes are also considered. Few enzyme-based methods that allow quantification of specific glycan structures, such as laminarin and α-glucans, in marine samples have been recently developed [13, 80]. However, due to the glycans’ structural complexity and diversity, the quantification of individual glycan types remains technologically challenging.

### Sandy sediments mineralize labile parts of photosynthesis-derived particulate organic matter and release more stable, glucose-depleted residual glycans

Marine dissolved organic matter (DOM) is the largest ocean reservoir of reduced carbon with ~662 Pg C [81]. Much of the porewater DOM originates from the deposited POM produced by primary production in surface waters [82]. While Svalbard sediments were rich in glucose (84% ± 14% of total glycans measured) and were similar to POM from other sites [83, 84], porewater showed a lower contribution of glucose (~15–25%; Supplementary Table S6), resulting in a more even distribution of the different monosaccharides. This is in line with previous findings for DOM composition in seawater (15% glucose) [85, 86] and porewater (average 28% glucose) [82, 87]. Together with our findings that the concentrations of monosaccharides in porewater were about one order of magnitude higher than in bottom water, these data suggest that benthic microbial communities transform OM, utilizing mostly glucose. Glucose-depleted DOM which is more stable against bacterial degradation is released into the water column by tidal pumping. Overall, we show that benthic microbiomes in sandy shelf sediments are major modulators of DOM composition, extending early findings by Burdige [87, 88] and Huettel and colleagues [89] who suggested that the sediments present a net source of dissolved organic carbon.

### Conclusion and outlook

Our data show that the majority of the benthic bacterial community in Svalbard is present and active in two contrasting seasons despite the strong seasonality in polar regions. These findings highlight that the bacterial communities of the water column and of underlying sediments respond differently to fresh OM input from algae blooms. Nevertheless, we found some seasonality, such as degradation of β-glucans by *Bacteroidia* and *Gammaproteobacteria* in spring, supporting our hypothesis that benthic bacterial communities respond to the seasonally changing input of fresh OM. Similar to what occurs in the water column, laminarin degradation is a major process in sediments during spring, while utilization of α-glucans, in particular glycogen, occurs throughout the year. The stable expression of genes for the degradation of other constantly available substrates, such as mucin and chitin, is consistent with our hypothesis that the continuous utilization of less labile, permanently available substrates stabilizes benthic bacterial communities.

Future studies could aim at the autecology of taxa degrading these often complex, permanently available substrates, for example, by enrichment and isolation of pure cultures using mucin and chitin. Yet, we hypothesize that it is the tremendous diversity on various trophic levels, the multiple niches, the complexity of substrates, and the highly dynamic conditions of coastal sandy sediments with currents and storms that make the benthic microbiome so robust and stable, both with respect to taxonomy and function. Benthic microbiomes thereby will remain an ultimate challenge for ecologists.

## Supplementary Material

Miksch_Supplementary_Information_wrad005

TabS5_allTPM_final_wrad005

TabS7_Translation_matrix_version_revised_wrad005

## Data Availability

Sequence data are available at ENA under the project accession PRJEB53193.
