# Supplementary material for: Taxonomic and functional stability overrules seasonality in polar benthic microbiomes
Source: ISME J. 2024 Jan 10;18(1):wrad005. doi: 10.1093/ismejo/wrad005 (PMC10811738; doi:10.1093/ismejo/wrad005)
Supplement: Miksch_Supplementary_Information_wrad005 [file miksch_supplementary_information_wrad005.pdf]

Supplementary Information

Taxonomic and functional stability overrules seasonality in polar benthic microbiomes

Sebastian Miksch[1], Luis H. Orellana[1], Monike Oggerin de Orube[1], Silvia Vidal-Melgosa[1,2], Vipul Solanki[1], Jan-Hendrik Hehemann[1,2], Rudolf Amann[1], Katrin Knittel[1*]

[1] Max Planck Institute for Marine Microbiology, Bremen, Germany

[2] MARUM, Center for Marine Environmental Sciences, University of Bremen, Bremen, Germany

Supplementary Materials and Methods

RNA extraction

RNA was extracted from sediment samples from Dec 2017, Feb 2018, and May 2018 from stations 5 and 7 using RNeasy PowerSoil Total RNA Kit (QIAGEN, Hilden, Germany) with some modifications to the manufacturer's recommendation. For May 2018 sediments, RNA was extracted from station 5 from three technical replicate samples.

Modifications were as follows:

- fresh phenol/chloroform/isoamyl alcohol (pH 6.5-8.0) was added to the tube containing the beads before adding the sample.
- As only up to 2.0 g of sample can be used per tube, we subsampled each sample, performing two to five extractions per sample. In particular for winter samples, several replicates were extracted and finally combined to retrieve an amount of RNA that is sufficiently high for metatranscriptomics.
- The incubation from step 7 of the manufacturer's protocol was performed on ice and the centrifugation was done at 3000 x g.
- The centrifugation speed used in step 10 was 5000 x g.
- The last incubation (step 17) was done at -20° C for 30 minutes.
- The centrifugation (step 18) was performed at 15000 x g for 30 minutes.
- For resuspension of the nucleic acid pellet, a smaller volume between 25 to 50 µl of SR7 solution was used.
- Contaminating DNA was digested by using the Invitrogen™ TURBO DNA-free™ Kit (Thermo Fisher Scientific, Bremen, Germany) following the manufacturers' protocol.

Sequence assembly and binning

Sequences were quality-controlled using BBTools v37.62 (quality < 20, minimum length 140 nt per read). Quality controlled reads were analyzed for their coverage of sequence diversity using nonpareil v3.303 [1]. Assembly of reads into contigs was done with SPAdes v3.13.1 [2] using k-mers 55, 77, 99, 127 and the meta option and assembly only. Assembly quality was evaluated using QUAST v4.5 [3]. Contigs < 1 kb length were excluded from further analyses. For each dataset, binning was done using MaxBin v2.2.7 [4] at default settings and using MetaBAT v2:2.15 [5] at default settings and –m 1500. Bin refinement was performed using DAS_Tool v1.1.2 at default settings [6]. Mapping for differential coverage binning was done using bbmap v38.70 and default settings [7] except for minid=0.99 and idfilter=0.97. De-replication of bins retrieved by DAS_Tool from all datasets was performed with Rep v3.1.1 [8] at default settings except for parameters –g 35, -l 1000, -comp 50, -con 15 and classified using the GTDB-Tk v2.1.1 [9] and GTDB release r214. Completeness and contamination was assessed in checkM v1.0.7 [10].

Gene annotation and analyses

Gene predictions from bins were done using Prokka v1.14.6 [11], dbCAN (run_dbCAN v2.0.11 workflow; https://github.com/linnabrown/run_dbcan) [12], Swiss-Prot release 2021_04 [13], SulfAtlas v1.0 [14], and transporterDB [download Oct21, 15]. The latter three databases were searched using DIAMOND blastp mode and default settings. Results were filtered for the best hit using the enveomics script BlastTab.best_hit_sorted [16] and >60% identity to reference sequences and a query coverage of >70%.

CAZyme annotations obtained from dbCAN were accepted when two of the three integrated annotation methods (HMMER v3.3.2, DIAMOND v2.0.9.147, Hotpep version included in run_dbCAN workflow) matched [12]. The annotations from Prokka and the databases (except

for dbCAN) were compared using a semi-automated approach. This comparison was done using a full Damerau-Levenshtein distance [17, 18] between the annotations from different tools/databases (0, identical string; 1,completely different). To account for discrepancies in the annotation string for identical functions in different databases (e.g. capitalization, extra spaces, extra letters), differences ≤25% of total string lengths were allowed. Results were sorted according to i) annotated function matching in at least two of the four databases, ii) only one database annotated the predicted gene (results marked with "*" at the end of the string) or iii), annotated function disagreed between databases (marked with "manualcheck_"). Metagenomic abundance was calculated as gene counts divided by genome equivalents that were estimated using MicrobeCensus [19].

Transcriptomic analyses

Quality-controlled RNA reads were sorted using SortMeRNA 4.0.4 [20] using default settings. Reads classified as rRNA were used for taxonomic profiling by using the SILVAngs pipeline [https://ngs.arb-silva.de/silvangs/, release 138.1, 21] and default settings. For expression analysis, all reads not classified as rRNA or tRNA, were considered as mRNA.

Annotation of transcripts was done by mapping mRNA to predicted genes from metagenomic contigs and bins using DIAMOND blastx [v2.0.15.153, 22] at default settings. Results were filtered for the best hit using the enveomics script BlastTab.best_hit_sorted [16] and >60% identity to reference sequences and a query coverage of >70%. Values of transcripts per million (TPM) mapped reads were calculated after normalization by gene length. Differences between seasons were calculated using the average TPM values in winter (December and February) vs. spring (May) and given as $\log_2$ fold change.

Data transformation and plotting was done using R and the tidyverse packages [23, 24].

Prediction of monosaccharide composition based on expression of GH genes.

For each GH family, all enzyme activities given in the CAZy database were extracted. Each enzyme activity is referred to one or several monosaccharide released/degraded. The number of different enzymes which can be referred to a specific monosaccharide is determined for each GH family and given in Supplementary Table S7. For the analysis, each enzyme activity given in the CAZy database is considered equally important. For example, for GH1, the following sugars are proposed based on 27 enzyme names: galactose (deduced from 3 enzyme names), glucose (deduced from 18 enzyme names), arabinose, fucose, glucuronic acid, rhamnose, mannose, and xylose (each deduced from 1 enzyme name). In a second step, TPM of expressed GHs were "translated" to the fraction a specific sugar account for by considering the relative contribution of this specific sugar to the GH family. For example, for GH1, release of glucose was predicted based on 18 out of 27 assignments, i.e. contributing 2/3 of total sugars. The final predicted monosaccharide composition is obtained by summing up the contributions of each sugars for each GH family. The script with detailed information is deposited on GitLab under https://gitlab.mpi-bremen.de/smiksch/gh_family_to_monosaccharides

Monosaccharide analysis

Freeze-dried sediment was homogenized and mixed with 5 ml Milli-Q ultrapure water per mg of sediment and incubated in a sonication bath (Bandelin Sonorex®, Berlin, Germany) for 60 min at maximum intensity. Afterwards, samples were centrifuged at 6000 x g for 15 min at 20° C and the supernatant was preserved. Porewater and OSW were dialyzed to remove salts using ~1 kDa mesh size dialysis bags (Spectra/Por®, Fisher Scientific, Bremen, Germany) for 24 h against Milli-Q water. Dialyzed samples were freeze-dried and re-suspended in a tenth of the

original volume. Both sediment extracts and pore water and overlying sea water samples were hydrolyzed using HCl (1 M final concentration) for 24 h. Monosaccharides were quantified using High performance anion exchange chromatography (HPAEC) with pulsed amperometric detection (PAD) for details see Vidal-Melgosa *et. al.* [25].

References

1. Rodriguez-R LM, Konstantinidis KT. Nonpareil: a redundancy-based approach to assess the level of coverage in metagenomic datasets. Bioinformatics. 2013;30:629-35.
2. Bankevich A, Nurk S, Antipov D, Gurevich AA, Dvorkin M, Kulikov AS, et al. SPAdes: A new genome assembly algorithm and its applications to single-cell sequencing. J Comput Biol. 2012;19:455-77.
3. Gurevich A, Saveliev V, Vyahhi N, Tesler G. QUAST: quality assessment tool for genome assemblies. Bioinformatics. 2013;29:1072-5.
4. Wu YW, Tang YH, Tringe SG, Simmons BA, Singer SW. MaxBin: an automated binning method to recover individual genomes from metagenomes using an expectation-maximization algorithm. Microbiome. 2014;2.
5. Kang DD, Li F, Kirton E, Thomas A, Egan R, An H, et al. MetaBAT 2: an adaptive binning algorithm for robust and efficient genome reconstruction from metagenome assemblies. PeerJ. 2019;7:e7359-e.
6. Sieber CMK, Probst AJ, Sharrar A, Thomas BC, Hess M, Tringe SG, et al. Recovery of genomes from metagenomes via a dereplication, aggregation and scoring strategy. Nature Microbiol. 2018;3:836-43.
7. Bushnell B, Rood J, Singer E. BBMerge - Accurate paired shotgun read merging via overlap. PLoS One. 2017;12:e0185056.
8. Olm MR, Brown CT, Brooks B, Banfield JF. dRep: a tool for fast and accurate genomic comparisons that enables improved genome recovery from metagenomes through de-replication. ISME J. 2017;11:2864-8.
9. Chaumeil P-A, Mussig AJ, Hugenholtz P, Parks DH. GTDB-Tk: a toolkit to classify genomes with the Genome Taxonomy Database. Bioinformatics. 2019;36:1925-7.
10. Parks DH, Imelfort M, Skennerton CT, Hugenholtz P, Tyson GW. CheckM: assessing the quality of microbial genomes recovered from isolates, single cells, and metagenomes. Genome Res. 2015;25:1043-55.
11. Seemann T. Prokka: rapid prokaryotic genome annotation. Bioinformatics. 2014;30:2068-9.

12. Huang L, Zhang H, Wu P, Entwistle S, Li X, Yohe T, et al. dbCAN-seq: a database of carbohydrate-active enzyme (CAZyme) sequence and annotation. Nucleic Acids Res. 2017;46:D516-D21.

13. Consortium TU. UniProt: the universal protein knowledgebase in 2021. Nucleic Acids Res. 2020;49:D480-D9.

14. Barbeyron T, Brillet-Guéguen L, Carré W, Carrière C, Caron C, Czjzek M, et al. Matching the diversity of sulfated biomolecules: creation of a classification database for sulfatases reflecting their substrate specificity. PLoS One. 2016;11:e0164846-e.

15. Elbourne LDH, Tetu SG, Hassan KA, Paulsen IT. TransportDB 2.0: a database for exploring membrane transporters in sequenced genomes from all domains of life. Nucleic Acids Res. 2016;45:D320-D4.

16. Rodriguez-R LM, Konstantinidis KT. The enveomics collection: a toolbox for specialized analyses of microbial genomes and metagenomes. PeerJ Preprints. 2016;4:e1900v1.

17. Levenshtein VI. Binary codes capable of correcting deletions, insertions, and reversals. Soviet Physics Doklady. 1966;10:707–10.

18. Damerau F. A technique for computer detection and correction of spelling errors. Communications of the ACM. 1964;7:171–6.

19. Nayfach S, Pollard KS. Average genome size estimation improves comparative metagenomics and sheds light on the functional ecology of the human microbiome. Genome Biology. 2015;16:51.

20. Kopylova E, Noé L, Touzet H. SortMeRNA: fast and accurate filtering of ribosomal RNAs in metatranscriptomic data. Bioinformatics. 2012;28:3211-7.

21. Quast C, Pruesse E, Yilmaz P, Gerken J, Schweer T, Yarza P, et al. The SILVA ribosomal RNA gene database project: improved data processing and web-based tools. Nucleic Acids Res. 2013;41:D590-D6.

22. Buchfink B, Reuter K, Drost H-G. Sensitive protein alignments at tree-of-life scale using DIAMOND. Nat Methods. 2021;18:366-8.

23. Team RC. R: A language and environment for statistical computing. Vienna, Austria: R Foundation for Statistical Computing, URL https://www.r-project.org/; 2019.

24. Wickham H, Averick M, Bryan J, Chang W, McGowan L, François R, et al. Welcome to the Tidyverse. J Open Source Softw. 2019;4:1686.

25. Vidal-Melgosa S, Sichert A, Francis TB, Bartosik D, Niggemann J, Wichels A, et al. Diatom fucan polysaccharide precipitates carbon during algal blooms. Nat Commun. 2021;12:1150.

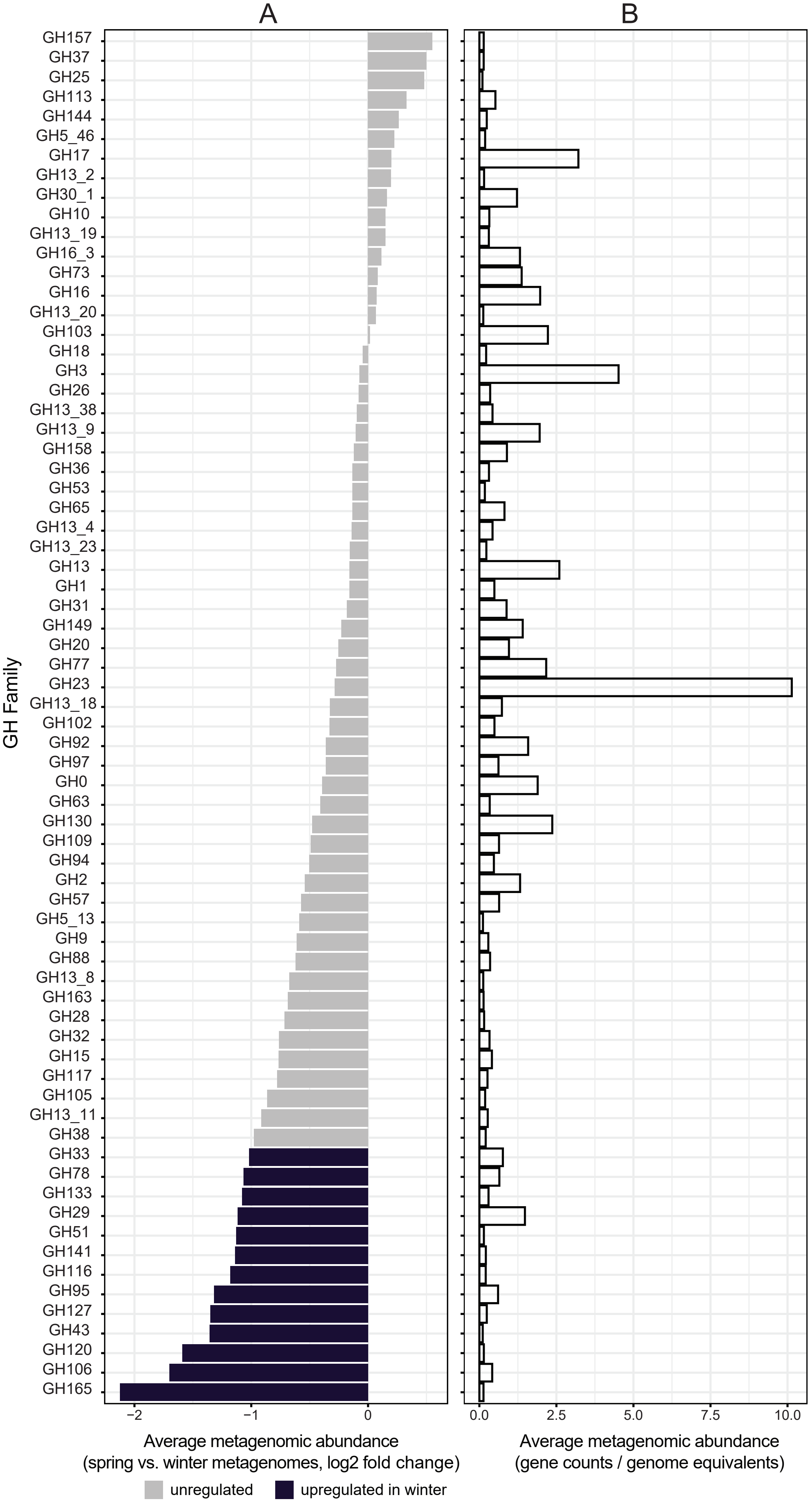

Supplementary Figure S1: Glycoside hydrolase families in the metagenomes. (A) Changes in average abundance of GH in spring metagenomes compared to winter metagenomes (calculated by abundance in spring / abundance in winter, given as log2 fold change), (B) average abundance in metagenomes (calculated by gene counts / genome equivalents estimated using MicrobeCensus) . Only GH families with > 0.1% average abundance are shown.

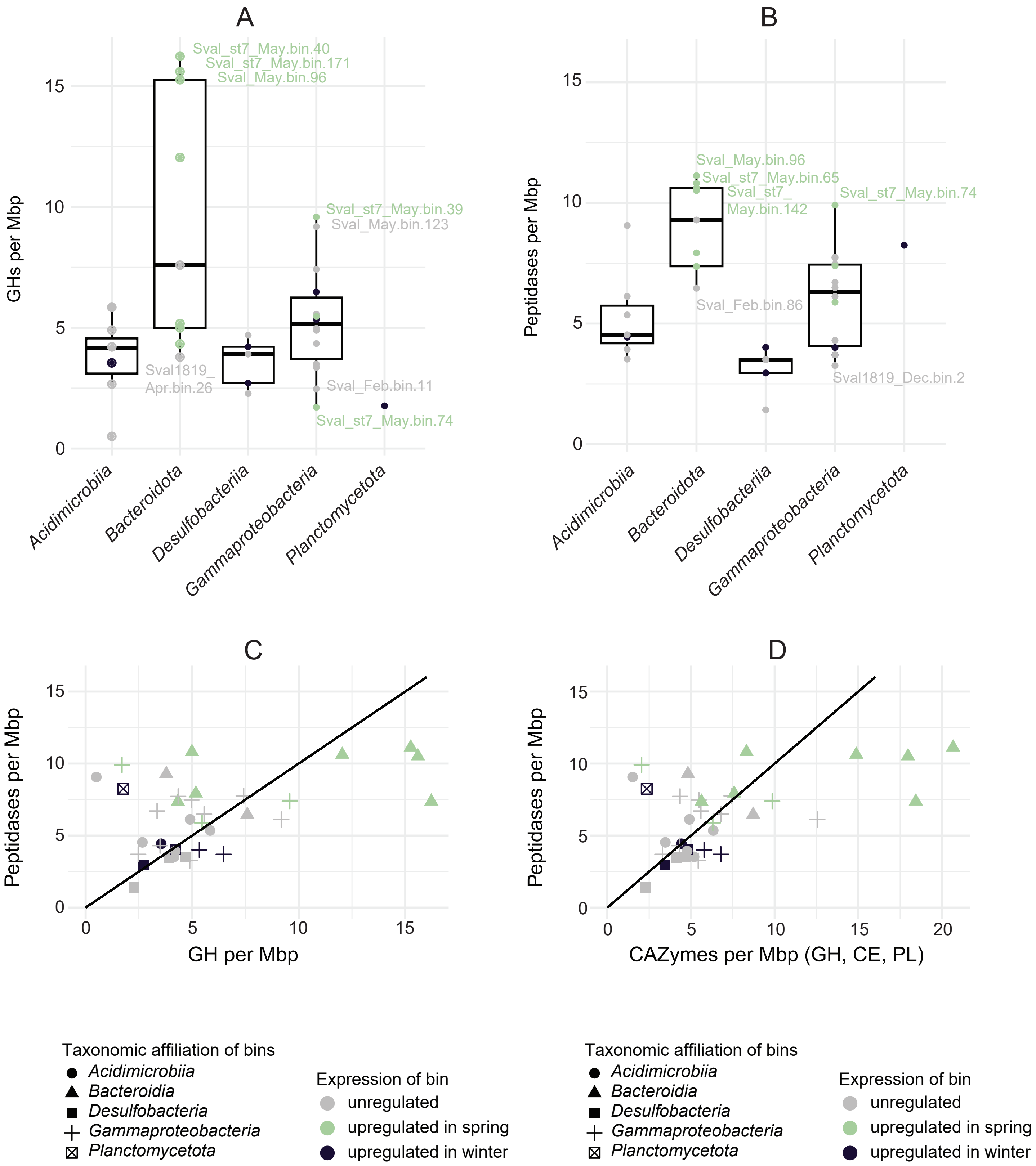

Supplementary Figure S2. Comparison of degradative CAZymes (GH +PL + CE) and peptidases repertoires in Svalbard *Acidimicrobiia, Bacteroidia*, *Gammaproteobacteria*, *Desulfobacteria,* and *Planctomycetota* bins. Abundance of (A) GH given per Mpb in bin, (B) peptidases, (C) GH versus peptidases to identify bins that represent specialists for carbohydrate degradation or protein degradation, and (D) CAZymes (sum of GH, CE, PL) versus peptidases. Gene abundances were normalized by bin size. GH, glycoside hydrolyses; CE, carbohydrate esterases, PL, polysaccharide lyases.

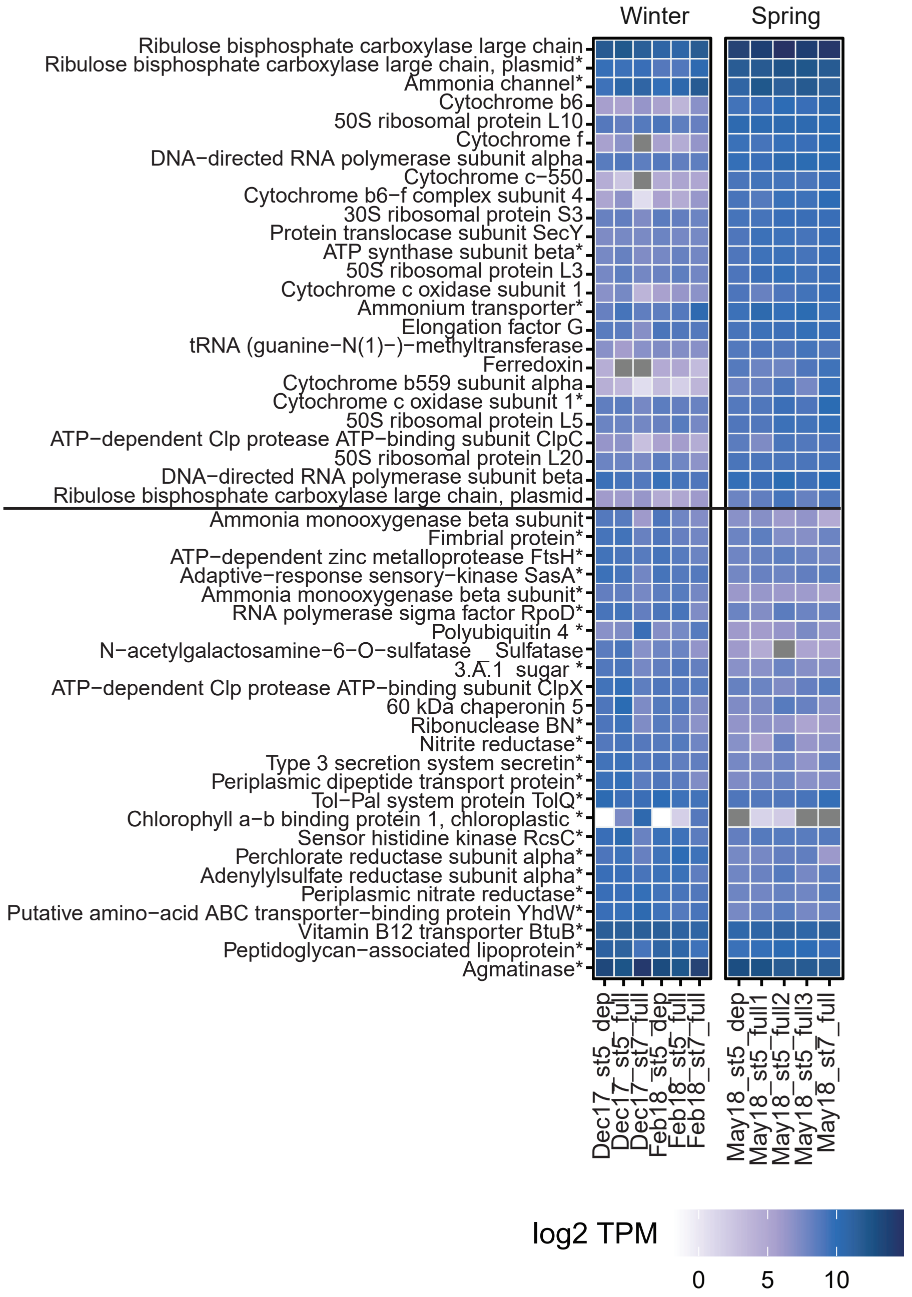

Supplementary Figure S3: Top 25 most expressed genes in winter (n=6) and spring (n=5) metatranscriptomes from Svalbard sediments [transcripts per million (TPM), given on a log2 scale]. Genes above the horizontal line are more expressed in spring while genes below are more expressed in winter. Genes, which could only be annotated by one of the used database, are marked by *. Grey tiles are infinite values after log transformation.

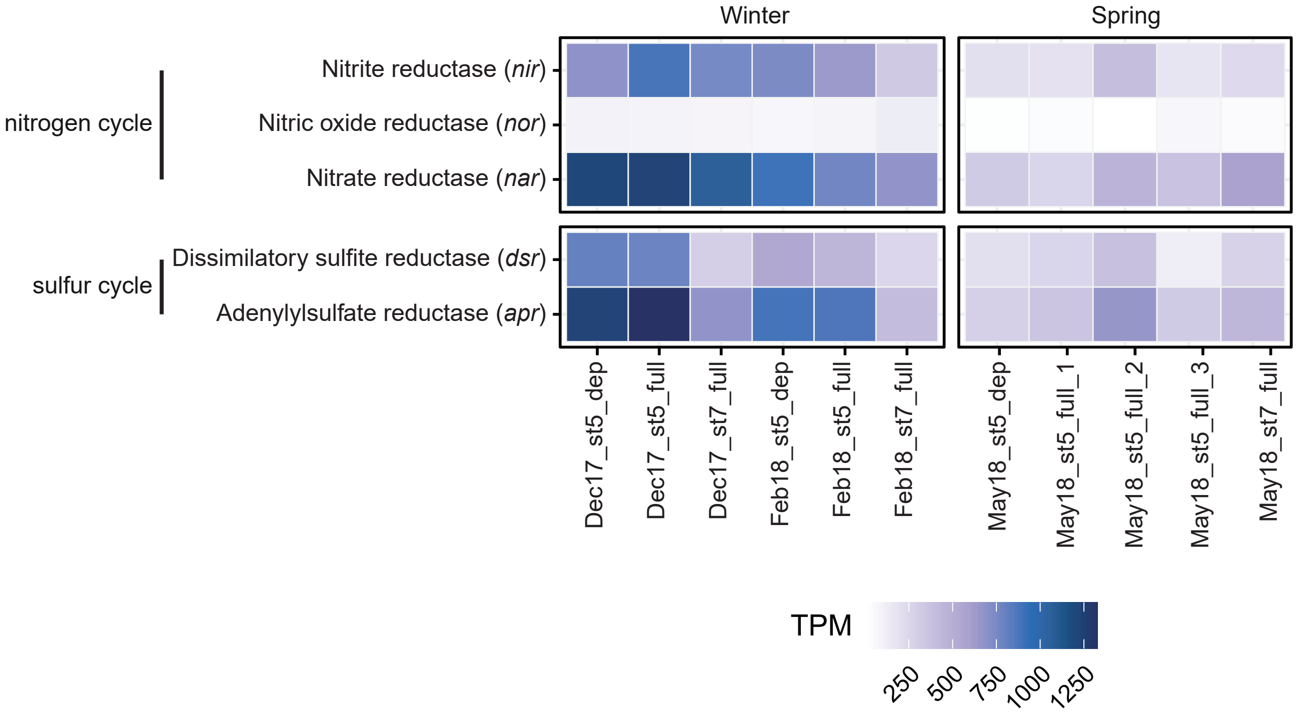

Supplementary Figure S4: Expression of key genes involved in nitrogen and sulfur cycling in Svalbard sediments. Abundances in winter and spring metatranscriptomes are shown in transcripts per million (TPM) reads. In particular, *nar* (nitrite reductase), *dsr* (dissimilatory sulfite reductase), and *apr* (adenylylsulfate reductase) were strongly upregulated in winter compared to spring.

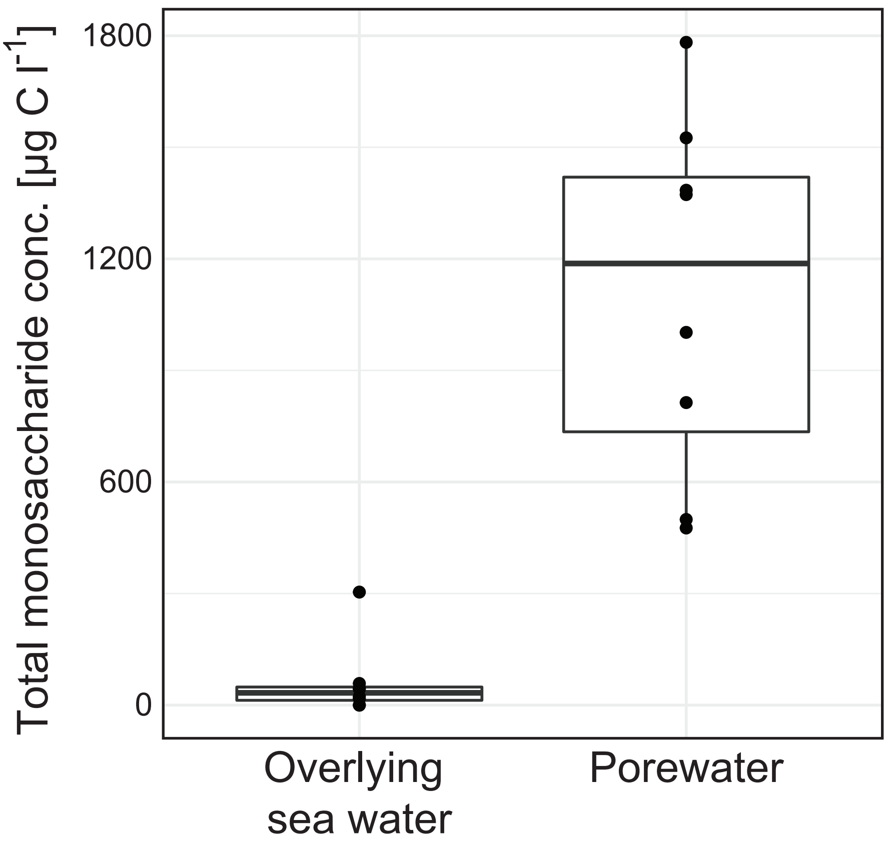

Supplementary Figure S5. Glycan concentrations in overlying seawater and porewater samples from Svalbard Isfjorden. Glycans from OSW and porewater were acid hydrolyzed and the resulting monosaccharides were measured by HPAEC-PAD analysis. Total monosaccharide concentration (sum of concentrations of all different monosaccharides) is shown. Concentrations in porewater were 18fold higher than in OSW. For concentrations of different monosaccharides refer to Supplementary Table S6.

A

Bin ID	reg.*	Contig ID	Genomic organization of CAZymes°	predicted substrate usage (www.cazy.org)
Sval_st7_May .bin.39	(green)	NODE_292	start of contig, …, GH30_1, CBM9, GH17, GH17, GH149, CBM57+GH16_3, GH17, GH16_3, GH16_3, CBM32+CBM56+CBM6	laminarin, β-glycans
		NODE_3883	start of contig, …, GH77, GH13+GH77+CBM48, GT35, CBM48+GH13_9	glycogen, α-glycans

*, reg., regulation. Bins upregulated in spring (green), assignment taken from Figure 3

° , PUL-like loci, based on the presence of ≥2 CAZyme within a 10-genes-sliding window
other CAZymes and CBM in contigs not matching the criteria and PUL-like loci: GH0+GH94+GT84, GH3, GH73, GH103, GH16_21, CBM50+GH23, GH23, GH13+CBM41+CBM48, GH42, GH13_18, GH3+GH30+CBM6, GH37, CE11

B

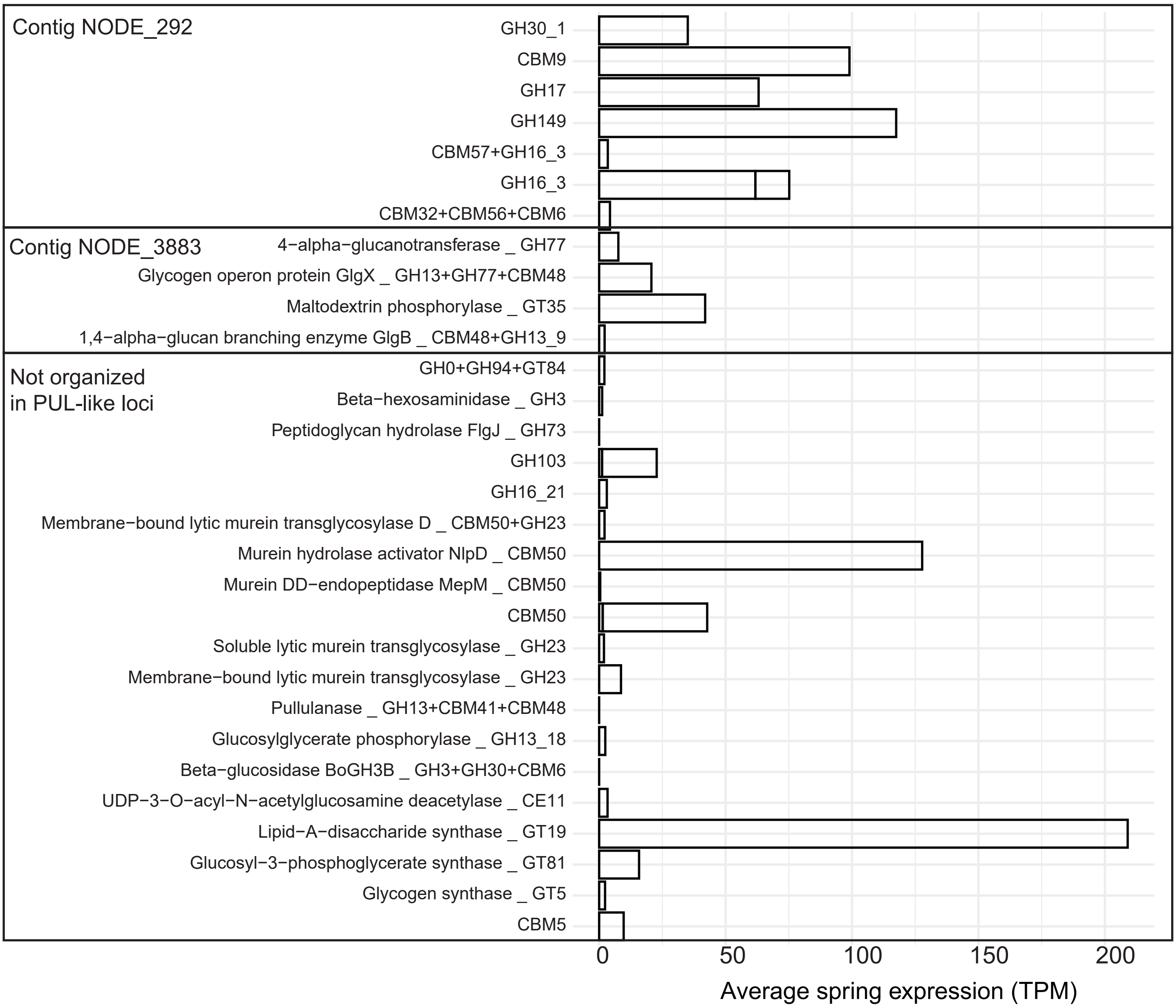

Supplementary Figure S6. Genomic organization (A) and expression of CAZymes (B) in Colwellia bin Sval_st7_May.bin39. A, In two contigs, PUL-like loci, defined by at least 2 CAZymes within a sliding window of 10 genes, were detected. Additionally, several other degradative CAZymes and CBM were found in the bin. B, Expression of genes from the two PUL-like structures in the spring metatranscriptomes. Stacked bars show the sum of expression of two different genes with identical annotations. Shown open reading frames/genes were not expressed in winter metatranscriptomes except for GH103 and GH73 with low expression levels of 0.2 TPM. GH73 are peptidoglycan hydrolases and slightly higher expressed than in spring metatrascriptomes.

Supplementary Table S1. Overview of samples from Isfjorden, Svalbard. OSW, overlying seawater; PW, porewater

sample ID	sample type	sampling date	station	metagenome	meta-transcriptome *full*	meta-transcriptome *rRNA depletion*	sugar analysis
Dec17_st5	bulk sediments	December 2017	5	x	x	x	x
Dec17_st7	bulk sediments	December 2017	7		x		x
Feb18_st5	bulk sediments	February 2018	5	x	x	x	x
Feb18_st7	bulk sediments	February 2018	7		x		x
May18_st5	bulk sediments	May 2018	5	x	xxx	x	x
May18_st7	bulk sediments	May 2018	7	x	x		x
Dec18_st5	bulk sediments	December 2018	5	x			x
Dec18_st7	bulk sediments	December 2018	7				x
Apr19_st5	bulk sediments	April 2019	5	x			x
Apr19_st7	bulk sediments	April 2019	7				x
Apr22_st5_7-bulk	bulk sediments	April 2022	5				x
Apr22_st5_8-bulk	bulk sediments	April 2022	5				x
Apr22_st5_11-bulk	bulk sediments	April 2022	5				x
Apr22_st5_12-bulk	bulk sediments	April 2022	5				x
Apr22_st5_7-PW	Porewater	April 2022	5				x
Apr22_st5_8-PW	Porewater	April 2022	5				x
Apr22_st5_11-PW	Porewater	April 2022	5				x
Apr22_st5_12-PW	Porewater	April 2022	5				x
Apr22_st5_7-OSW	OSW	April 2022	5				x
Apr22_st5_8-OSW	OSW	April 2022	5				x
Apr22_st5_11-OSW	OSW	April 2022	5				x
Apr22_st5_12-OSW	OSW	April 2022	5				x

Supplementary Table S2: Basic statistics for 6 metagenomic and 11 metatranscriptomic datasets obtained from Svalbard sediments.

dataset ID	type		no. reads (raw reads)	no reads (after qc)	[%] left after quality control	redundancy (nonpareil)	contigs (>1kb)	N50	bins	reads (mRNA)
Sval_Dec_2017	metagenome		139,473,476	103,802,669	74.42	0.49	973,058	1,781	34	-
Sval_Feb_2018	metagenome		125,891,034	107,222,317	85.17	0.49	1,013,048	1,859	35	-
Sval_May_2018_st5	metagenome		133,679,990	115,909,120	86.71	0.47	1,084,287	1,765	40	-
Sval_May_2018_st7	metagenome		135,845,280	118,392,707	87.15	0.45	1,026,676	1,834	42	-
Sval_Dec_2018	metagenome		98,660,349	98,109,189	99.44	0.46	671,159	1,718	16	-
Sval_Apr_ 2019	metagenome		91,594,369	91,031,268	99.39	0.50	562,393	1,605	16	-
Sval_Dec_st5_dep	metatranscriptome	rRNA depletion	104,435,692	90,587,256	86.74	-	-	-	-	72,961,714
Sval_Dec_st5_full	metatranscriptome		160,797,588	117,796,866	73.26	-	-	-	-	3,608,598
Sval_Dec_st7_full	metatranscriptome		173,253,912	156,932,902	90.58	-	-	-	-	7,688,798
Sval_Feb_st5_dep	metatranscriptome	rRNA depletion	84,267,052	55,112,272	65.40	-	-	-	-	44,000,378
Sval_Feb_st5_full	metatranscriptome		162,431,412	118,600,342	73.02	-	-	-	-	3,556,094
Sval_Feb_st7_full	metatranscriptome		163,666,564	144,462,378	88.27	-	-	-	-	4,103,232
Sval_May_st5_dep	metatranscriptome	rRNA depletion	96,831,938	87,716,354	90.59	-	-	-	-	54,005,690
Sval_May_st7_full	metatranscriptome		178,072,220	166,552,672	93.53	-	-	-	-	4,082,580
Sval_May1 st5_full	metatranscriptome		157,004,704	127,115,722	80.96	-	-	-	-	3,871,750
Sval_May2_st5_full	metatranscriptome		178,707,738	156,811,100	87.75	-	-	-	-	2,038,998
Sval_May3_st5_full	metatranscriptome		186,663,216	169,326,994	90.71	-	-	-	-	4,500,940

Supplementary Table S3: Statistics of high-quality bins.

Bin ID	Taxonomic classification	Completeness [%]	Contamination [%]	Genome size [bp]	contigs [no.]	N50 [bp]	16S rRNA gene [yes/no]
Sval_Feb.bin.32	d__Bacteria;p__Actinomycetota;c__Acidimicrobiia;o__Acidimicrobiales;f__Ilumatobacteraceae;g__Ilumatobacter_A;s__	92.5	8.12	4,821,387	876	6,911	no
Sval_st7_May.bin.207	d__Bacteria;p__Actinomycetota;c__Acidimicrobiia;o__Acidimicrobiales;f__Ilumatobacteraceae;g__Ilumatobacter_A;s__	85.5	5.13	3,751,020	839	5,208	no
Sval_st7_May.bin.7	d__Bacteria;p__Actinomycetota;c__Acidimicrobiia;o__Acidimicrobiales;f__SZUA-35;g__;s__	87.1	5.59	3,564,160	362	14,464	yes
Sval_May.bin.58	d__Bacteria;p__Actinomycetota;c__Acidimicrobiia;o__UBA5794;f__JAENVV01;g__;s__	78.2	5.98	1,985,680	347	7,626	yes
Sval_Dec.bin.7	d__Bacteria;p__Actinomycetota;c__Acidimicrobiia;o__UBA5794;f__UBA4744;g__UBA4744;s__	65.4	9.13	2,053,696	524	5,023	no
Sval_May.bin.130	d__Bacteria;p__Actinomycetota;c__Acidimicrobiia;o__UBA5794;f__UBA5794;g__JAHEEL01;s__	87.6	3.94	2,448,020	490	6,341	yes
Sval_Dec.bin.66	d__Bacteria;p__Actinomycetota;c__Acidimicrobiia;o__UBA5794;f__UBA5794;g__B3-G11;s__	87.3	4.89	2,256,330	599	4,231	yes
Sval_Feb.bin.86	d__Bacteria;p__Bacteroidota;c__Bacteroidia;o__Flavobacteriales;f__JAHECK01;g__JAHECK01;s__	89.0	9.21	3,558,031	741	6,250	no
Sval_st7_May.bin.218	d__Bacteria;p__Bacteroidota;c__Bacteroidia;o__Flavobacteriales;f__Crocinitomicaceae;g__UBA4466;s__	78.5	5.15	2,310,277	501	5,574	no
Sval1819_Apr.bin.26	d__Bacteria;p__Bacteroidota;c__Bacteroidia;o__Flavobacteriales;f__Crocinitomicaceae;g__UBA4466;s__	94.7	1.80	2,906,039	366	12,393	yes
Sval_st7_May.bin.35	d__Bacteria;p__Bacteroidota;c__Bacteroidia;o__Flavobacteriales;f__Flavobacteriaceae;g__GCA-2733415;s__	97.8	2.34	2,900,808	175	26,888	no
Sval_st7_May.bin.65	d__Bacteria;p__Bacteroidota;c__Bacteroidia;o__Flavobacteriales;f__Flavobacteriaceae;g__GCA-2733415;s__	97.4	1.26	2,404,623	91	69,552	no
Sval_May.bin.96	d__Bacteria;p__Bacteroidota;c__Bacteroidia;o__Flavobacteriales;f__Flavobacteriaceae;g__Lutimonas;s__	92.7	1.15	3,145,817	231	21,458	no
Sval_st7_May.bin.171	d__Bacteria;p__Bacteroidota;c__Bacteroidia;o__Flavobacteriales;f__Flavobacteriaceae;g__Lutimonas;s__	83.7	4.57	2,949,660	371	11,197	no
Sval_st7_May.bin.40	d__Bacteria;p__Bacteroidota;c__Bacteroidia;o__Flavobacteriales;f__Flavobacteriaceae;g__SCGC-AAA160-P02;s__	95.1	1.79	2,712,007	169	26,245	no
Sval_st7_May.bin.142	d__Bacteria;p__Bacteroidota;c__Bacteroidia;o__Flavobacteriales;f__Flavobacteriaceae;g__SHLJ01;s__	93.0	0.99	2,823,433	186	23,121	no
Sval_Dec.bin.161	d__Bacteria;p__Desulfobacterota;c__Desulfobacteria;o__Desulfobacterales;f__JAFDCJ01;g__JAFDCJ01;s__	83.2	0.97	4,064,951	581	9,140	no
Sval_Feb.bin.57	d__Bacteria;p__Desulfobacterota;c__Desulfobacteria;o__Desulfobacterales;f__JAFDCJ01;g__JAFDCJ01;s__	53.7	2.61	3,516,276	904	4,227	no
Sval_Feb.bin.151	d__Bacteria;p__Desulfobacterota;c__Desulfobacteria;o__Desulfobacterales;f__Desulfosarcinaceae;g__Desulfosarcina;s__	76.9	5.18	4,266,660	1,195	3,908	no
Sval_Feb.bin.47	d__Bacteria;p__Desulfobacterota;c__Desulfobacteria;o__Desulfobacterales;f__Desulfosarcinaceae;g__Desulfosarcina;s__	91.6	1.79	4,984,765	655	11,167	no
Sval_Dec.bin.130	d__Bacteria;p__Desulfobacterota;c__Desulfobulbia;o__Desulfobulbales;f__Desulfocapsaceae;g__MADRE3;s__	92.4	1.92	4,863,787	827	7,810	no
Sval_Feb.bin.76	d__Bacteria;p__Planctomycetota;c__UBA1135;o__UBA1135;f__UBA1135;g__;s__	67.4	2.25	1,697,842	558	3,225	no
Sval_st7_May.bin.39	d__Bacteria;p__Proteobacteria;c__Gammaproteobacteria;o__Enterobacterales;f__Alteromonadaceae;g__Colwellia_A;s__	95.9	1.92	3,653,294	194	28,410	no
Sval_Feb.bin.11	d__Bacteria;p__Proteobacteria;c__Gammaproteobacteria;o__GCA-001735895;f__GCA-001735895;g__GCA-001735895;s__	93.5	9.01	4,860,664	484	16,021	yes
Sval_Feb.bin.110	d__Bacteria;p__Proteobacteria;c__Gammaproteobacteria;o__GCA-001735895;f__GCA-001735895;g__GCA-001735895;s__	82.8	7.27	3,718,874	717	6,494	no
Sval_st7_May.bin.17	d__Bacteria;p__Pseudomonadota;c__Gammaproteobacteria;o__Pseudomonadales;f__Porticoccaceae;g__HTCC2207;s__HTCC2207 sp905182275	91.6	4.01	2,378,634	381	8,914	yes
Sval_Dec.bin.80	d__Bacteria;p__Pseudomonadota;c__Gammaproteobacteria;o__UBA9214;f__UBA9214;g__;s__	84.2	14.69	3,241,495	605	7,338	no
Sval_Feb.bin.103	d__Bacteria;p__Pseudomonadota;c__Gammaproteobacteria;o__UBA9214;f__UBA9214;g__UBA9214;s__	78.1	5.66	2,247,594	530	4,764	no
Sval_Dec.bin.61	d__Bacteria;p__Pseudomonadota;c__Gammaproteobacteria;o__Woeseiales;f__Woeseiaceae;g__JAACFB01;s__	79.7	9.77	2,683,288	246	13,937	no
Sval_Dec.bin.29	d__Bacteria;p__Pseudomonadota;c__Gammaproteobacteria;o__Woeseiales;f__Woeseiaceae;g__UBA1847;s__	84.0	3.02	3,236,586	134	39,867	yes
Sval_May.bin.123	d__Bacteria;p__Pseudomonadota;c__Gammaproteobacteria;o__Woeseiales;f__Woeseiaceae;g__UBA1847;s__	89.0	0.79	3,267,154	127	42,481	no
Sval_May.bin.64	d__Bacteria;p__Pseudomonadota;c__Gammaproteobacteria;o__Woeseiales;f__Woeseiaceae;g__UBA1847;s__	75.3	6.90	2,965,941	443	8,357	no
Sval_st7_May.bin.4	d__Bacteria;p__Pseudomonadota;c__Gammaproteobacteria;o__Woeseiales;f__Woeseiaceae;g__UBA1847;s__	79.0	2.32	2,071,716	205	12,561	no
Sval_st7_May.bin.74	d__Bacteria;p__Pseudomonadota;c__Gammaproteobacteria;o__Xanthomonadales;f__Marinicellaceae;g__NORP309;s__	89.7	6.01	2,926,992	244	18,992	no
Sval_st7_May.bin.147	d__Bacteria;p__Pseudomonadota;c__Gammaproteobacteria;o__Xanthomonadales;f__SZUA-36;g__JAJRRA01;s__	90.9	8.92	4,020,067	773	6,499	yes
Sval1819_Dec.bin.2	d__Bacteria;p__Pseudomonadota;c__Gammaproteobacteria;o__Xanthomonadales;f__SZUA-36;g__SZUA-36;s__	63.8	2.54	1,841,078	521	3,923	no

Supplementary Table S4. Genomic organization of CAZymes in Bacteroidia bins. +, genes within the same open reading frame.

bin ID	reg.*	contig ID	Genomic organization of CAZymes as PUL or PUL-like structures♦			predicted substrate usage (www.cazy.org)	other degradative CAZymes and CBM• (not organized in PUL-like structures)
Sval_st7_May.bin.40		NODE_216	Start of contig, …,	SusCD	CBM4+CBM4,GH16_3, GH2, GH149, GH30_1, GH17, …, end	laminarin, β-glycans	GH16+CBM54, GH3, GH130, GH13_31, GH43_12, GH92, GH81+CBM6, GH3, GH109, GH39, GH125, GH23+CBM50, GH20+CBM32, GH0+GH113, CE11, CE1, CE14, CE4
		NODE_59	start of contig, …, GH5_4, GH3, GH10, GH10, GH43_1, GH10, PL9+CBM22, GH30,	SusCD	…, end of contig	xylane, xylose	
		NODE_650	start of contig, ..., GH92, GH92+GH94, GH92,	SusC	…, end of contig	mannose	
		NODE_711	start of contig- GH30_1, GH16, GH5_46+CBM6+CBM6,	SusC	…, end of contig	β-glycans	
		NODE_848	start of contig,…,	SusC	GH92, GH92, GH92+GH94, GH3, GH3+CBM6,…, end of contig	mannose	
Sval_st7_May.bin.65		NODE_1389	start of contig, …, PL7_5, PL6_1, PL6+CBM32, PL17_2+PL17,	SusCD	, …, end of contig	alginate	GH23, GH113, GH25, CBM50+GH23, GH3, GH5_42, GH73+CBM50, CBM32+GH85, GH88, CE11, CE14, CE4,
		NODE_424	start of contig, GH10, GH3,	SusCD	…, end of contig	xylane, xylose	
Sval_st7_May.bin.218		NODE_2898	start of contig, …,	SusCD	GH16+CBM13+CBM6, end of contig	β-glycans	CBM50+GH23, GH3, GH92, GH2, GH1, GH113, GH23, GH20, CE14, CE1, CE4
Sval_st7_May.bin.35		NODE_30472	Start of contig-GH95+CBM13+CBM32+CBM51, GH168, GH30_2,	-	…, end of contig	fucose, fucan, xylane	GH3, GH157, GH20, GH25, GH18, CBM32+GH85, GH29, GH113, GH3, GH23, GH5_42, GH157, CBM50+GH23, CE1, CE11, CE14, CE4, PL6+PL6_1
Sval_st7_May.bin.142		NODE_649	start of contig, …, GH30_1, GH16+CBM4+CBM54,	SusCD	…, end of contig	laminarin, β-glycans	GH13, GH31, GH97, GH113, GH23, CBM48+GH13_9, GH0, GH30_1, GH17, CBM48+GH13, GH3, GH16+CBM4, GH2+CBM32+CBM67, GH53, CBM50+GH23, GH73+CBM50, GH158, CE11, CE14
		NODE_2327	start of contig, …, PL7,	SusCD	-PL17_2- … - PL7_5- …- end of contig	alginate	
		NODE_10	start of contig, …, GH92, GH20+CBM32, GH2, CE4,	-	…, end of contig	mannose, peptidoglycan	
		NODE_230	start of contig-GH13, GH31, GH13_19, GH97, GH13,	-	…, end of contig	α-glycans	
		NODE_29972	start of contig- …, GH17, GH17, GH17,	-	end of contig	laminarin, β-glycans	
		NODE_56	start of contig, ...-GH2+CBM51+CBM67, GH2+CBM51+CBM67, GH63,	-	…, end of contig	mannose	
		NODE_5907	start- GH158, GH0, GH16+CBM11+CBM13+CBM32+CBM4+CBM54+CBM56+CBM6, CBM4, GH30_1,	-	end of contig	β-glycans	
Sval_st7_May.bin.171		NODE_9839	Start of contig- PL17_2,	SusCD	…, end of contig	alginate	GH17, GH3, GH94, GH0+GH13, CE1+GH10, GH20+CBM32, GH149, GH144, GH5_48, GH23, GH20, GH13+CBM25+CBM41+CBM48+CBM68, CBM50+GH23, GH73+CBM50, GH3+CBM6, GH16, GH5_46+CBM6+CBM6, GH109, GH2+CBM32+CBM67, GH157, GH29, GH2, GH53, GH130, GH26, PL7, PL7_5, PL12, CE1, CE11, CE14
		NODE_1447	Start of contig,	SusCD	GH16_3, …, end of contig	laminarin, β-glycans	
		NODE_1541	Start of contig-GH13_19, GH13, GH65,	SusC	CBM48+GH13, …, end of contig	α-glycans	
		NODE_12460	Start of contig, …, GH16_3, GH17, GH17, GH17,	-	end of contig	laminarin, β-glycans	
		NODE_8488	Start of contig- GH158, GH17, GH149, GH30_1,	-	end of contig	laminarin, β-glycans	
Sval_May.bin.96		NODE_14	start of contig, …, CBM48+GH13,	SusCD	, GH65, GH13, GH31, GH13_19, GH13_38, …, end of contig	glycogen, α-glycans	GH20, GH3, GH5_46+CBM6+CBM6, GH26, GH3, GH9+CBM2+CBM4, GH0+GH13, GH23, CBM48+GH13_9, GH31, GH94, GH73+CBM50, GH109, GH0, GH2+CBM51+CBM67, GH95+CBM13+CBM51, GH16, GH130, GH16+CBM13+CBM32+CBM4+CBM54+CBM56+ CBM6, GH29, GH3+CBM6, CE4, CE14, CE11, PL6+PL6_1
		NODE_2396	start of contig- PL17_2,	SusCD	, PL7, PL7, …, end of contig	alginate	
		NODE_309	start of contig, …, PL7, PL7,	SusCD	, PL7_5, PL7_5, PL12, PL6+PL6_1, …, end of contig	alginate	
		NODE_2289	start of contig, …, GH94, GH16_3, GH17, GH17, GH17,	-	end of contig	laminarin, β-glycans	
		NODE_25	start of contig, …, GH20, GH3, GH3,	-	…, end of contig	xylose, peptidoglycan	
		NODE_9324	start of contig, …, GH16+CBM4, GH16+CBM4+CBM54+CBM6, GH30_1,	-	…, end of contig	laminarin, β-glycans	
Sval1819_Apr.bin.26				-		-	GH20+CBM32, GH73+CBM50, GH113, GH16+CBM11+CBM13, GH23, GH25, GH3, GH2, GH92, GH74, CE11, CE14, CE4
Sval_Feb.bin.86				-		-	GH130, CBM48+GH13, GH31, GH13_23, GH2+ CBM51+CBM67, GH73, GH158, GH23, GH38, GH65, GH29, GH2, GH92, CBM50+GH23, GH13, GH3, GH63, GH20, GH23, GH76, CE14, CE15, CE11, CE4

reg., regulation. Color indicates if bins were upregulated in spring (green) or were unregulated and evenly expressed throughout the year (grey; assignment taken from Figure 3)

♦, PUL are defined based on the presence of a pair of susC- and susD-like transporter genes and≥2 CAZyme genes (GHs, PLs, CEs or CBMs) and PUL-like loci based on the presence of ≥ 1 CAZyme, both within a 10-genes-sliding window).

•, other CAZymes in contigs not matching the criteria of PUL and PUL-like

Supplementary Table S6. Glycan concentrations in porewater (PW) and overlying seawater (OSW) from Isforden, Svalbard. Glycans from OSW and porewater were acid hydrolyzed and the resulting monosaccharides were measured by HPAEC-PAD analysis. Concentrations in PW were 18fold higher than in OSW. Values in surface seawater were in the range obtained from controls with demineralized water in dialysis bags (data not shown). Bd, below detection; bt, below threshold (values measured for low-concentrated calibration standards indicated a loss of detector sensitivity with time, thus a threshold concentration for each monosaccharide was set to the value at which the variation between two injections was smaller than ±20%. Values lower than the threshold concentrations defined for each monosaccharide were rejected).

ID	Replicate	Sample type	Arabinose [µg C L^{-1}]	Fucose [µg C L^{-1}]	Galactosamine [µg C L^{-1}]	Galactose [µg C L^{-1}]	Glucosamine [µg C L^{-1}]	Glucose [µg C L^{-1}]	Mannose [µg C L^{-1}]	Rhamnose [µg C L^{-1}]	Xylose [µg C L^{-1}]	Sum [µg C L^{-1}]
Apr_7-OSW	1	OSW	bd	bd	bt	bt	bt	bt	bt	bd	bt	0.0
Apr_7-OSW	2	OSW	bd	bd	bt	bt	bt	bt	bt	bd	bt	0.0
Apr_8-OSW	1	OSW	bd	bt	bt	bt	bt	22.9	bt	bd	bt	22.9
Apr_8-OSW	2	OSW	bd	bt	bt	bt	bt	17.2	bt	bd	bt	17.2
Apr_11-OSW	1	OSW	bd	bt	bt	bt	bt	43.2	bd	bd	bt	43.2
Apr_11-OSW	2	OSW	bd	bt	bt	bt	bt	45.5	bt	bd	bt	45.5
Apr_12-OSW	1	OSW	bt	bt	bt	bt	bt	58.2	bt	bd	bt	58.2
Apr_12-OSW	2	OSW	bt	bt	bt	14.4	bt	151.2	44.8	bd	95.0	305.4
Apr_7-PW	1	PW	103.8	79.4	bt	46.8	52.1	112.0	bt	bt	105.2	499.2
Apr_7-PW	2	PW	89.2	75.8	bt	43.6	51.6	125.2	bt	bt	90.9	476.3
Apr_8-PW	1	PW	bt	186.6	41.9	96.9	128.5	151.9	bt	bt	207.8	813.6
Apr_8-PW	2	PW	190.9	196.0	bt	100.7	144.0	163.7	bt	bt	206.9	1,002.3
Apr_11-PW	1	PW	399.4	365.4	11.2	192.8	248.7	238.7	bt	bt	326.0	1,782.2
Apr_11-PW	2	PW	99.5	349.3	83.5	186.6	247.3	240.4	bt	bt	318.8	1,525.4
Apr_12-PW	1	PW	262.9	289.4	10.1	148.9	179.2	198.9	bt	bt	283.4	1,372.8
Apr_12-PW	2	PW	269.5	303.1	bt	148.9	176.7	190.5	bt	bt	295.8	1,384.5
